# Fluorescent bioinspired albumin/polydopamine nanoparticles and their interactions with *Escherichia coli* cells

**DOI:** 10.3762/bjnano.14.100

**Published:** 2023-12-22

**Authors:** Eloïse Equy, Jordana Hirtzel, Sophie Hellé, Béatrice Heurtault, Eric Mathieu, Morgane Rabineau, Vincent Ball, Lydie Ploux

**Affiliations:** 1 UMR_S1121, INSERM/University of Strasbourg, 1 rue Eugène Boeckel, 67084 Strasbourg, France; 2 Faculty of Dentistry, University of Strasbourg, 8 Rue Ste Elisabeth, 67000 Strasbourg, France; 3 UMR 7199, CNRS/University of Strasbourg, 74 route du Rhin, 67401 Illkirch, France; 4 CNRS, 23 rue du Loess, 67200 Strasbourg, France

**Keywords:** accumulation, albumin, antibacterial, *Escherichia coli*, fluorescence, nanoparticles, penetration, polydopamine

## Abstract

Inspired by the eumelanin aggregates in human skin, polydopamine nanoparticles (PDA NPs) are promising nanovectors for biomedical applications, especially because of their biocompatibility. We synthesized and characterized fluorescent PDA NPs of 10–25 nm diameter based on a protein containing a lysine–glutamate diad (bovine serum albumin, BSA) and determined whether they can penetrate and accumulate in bacterial cells to serve as a marker or drug nanocarrier. Three fluorescent PDA NPs were designed to allow for tracking in three different wavelength ranges by oxidizing BSA/PDA NPs (Ox-BSA/PDA NPs) or labelling with fluorescein 5-isothiocyanate (FITC-BSA/PDA NPs) or rhodamine B isothiocyanate (RhBITC-BSA/PDA NPs). FITC-BSA/PDA NPs and RhBITC-BSA/PDA NPs penetrated and accumulated in both cell wall and inner compartments of *Escherichia coli* (*E. coli*) cells. The fluorescence signals were diffuse or displayed aggregate-like patterns with both labelled NPs and free dyes. RhBITC-BSA/PDA NPs led to the most intense fluorescence in cells. Penetration and accumulation of NPs was not accompanied by a bactericidal or inhibitory effect of growth as demonstrated with the Gram-negative *E. coli* species and confirmed with a Gram-positive bacterial species (*Staphylococcus aureus*). Altogether, these results allow us to envisage the use of labelled BSA/PDA NPs to track bacteria and carry drugs in the core of bacterial cells.

## Introduction

Organic nanoparticles (ONPs) are used to target and deliver drugs to tissue and eukaryotic cells [1–2], or bacteria and biofilms [3–4]. As nanovectors of drugs, they can deliver drugs locally, leading to a more efficient drug activity. Also, the required doses and the drug impact on healthy tissues compared to the free drug are lowered. Regarding the dramatic emergence and spreading of antimicrobial resistance of bacteria [5], this appears as a promising route to deliver antimicrobials while reducing the drug doses and subsequent harmful side effects in antibacterial applications. To this end, different types of ONPs have been used, such as liposomes [6] and nanoparticles (NPs) of poly(lactic-*co*-glycolic acid) (PLGA) [7], polycaprolactone [8], and chitosan [9]. Furthermore, fluorescent ONPs are a promising way to facilitate the localization of NPs in cells through fluorescence imaging. They can also be used for fluorescent labelling of cells, especially for live cell imaging, provided that the ONPs are harmless for cells. This has been developed for eukaryotic cells [10], but the use of ONPs for labelling bacterial cells is still rare and not described in literature for alive bacterial cells. The main limitation is probably the frequent cytotoxic effect of ONPs on bacteria.

**Figure 1:**
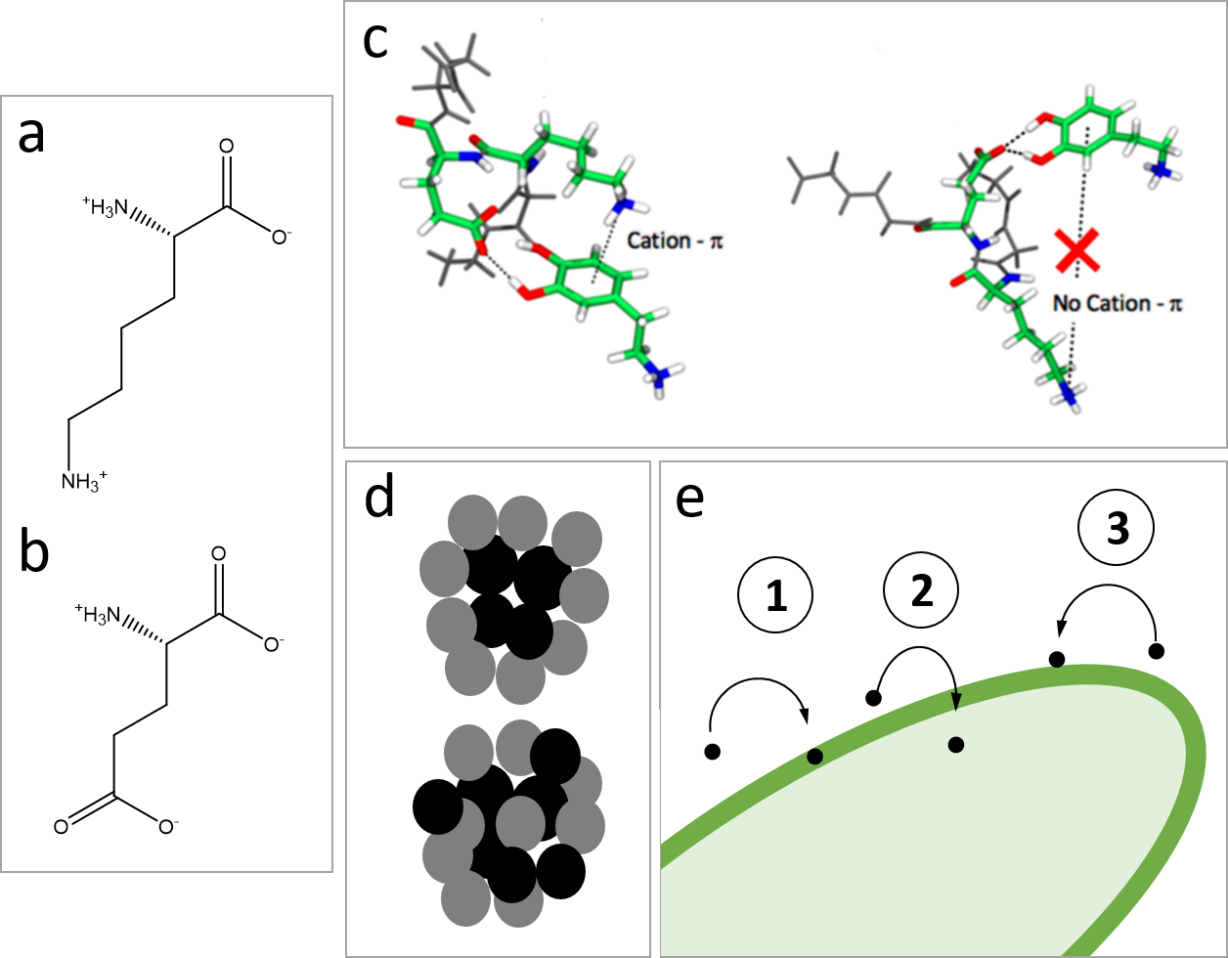
Molecules of (a) lysine (K) and (b) glutamate (E). (c) Interactions between dopamine and KE sequence. (d) Possible structures of protein (grey)/polydopamine (black) composite particles (inspired from [14]). (e) Possible locations of organic nanoparticles in bacterial cells. Figure 1c was adapted with permission from [13]. Copyright 2018 American Chemical Society. This content is not subject to CC BY 4.0.

Inspired by the eumelanin aggregates in human skin, polydopamine nanoaggregates (here referred to as nanoparticles, i.e., PDA NPs) have emerged as promising nanovectors for biomedical applications [11–12], especially because of their biocompatibility [13–14] and photothermic properties [15–16]. These properties can even be controlled by an external signal [17–19]. PDA NPs are formed upon oxidation in dopamine (DA) solutions with additives such as surfactants, polyelectrolytes, and proteins [14]. Eumelanin-like NPs with a diameter less than 20 nm have been obtained by this method. The additive plays a crucial role in the control of the NP size. Specifically, Bergtold et al. demonstrated that a protein (e.g. chromofungin) containing a diad of lysine (K) and glutamate (E) (Figure 1a,b) in its sequence allows for the control of the formation of PDA NPs, in contrast to an additive without a KE diad (e.g., catestatin) [13]. During the formation process, hydroxy groups of dopamine form hydrogen bonds with carboxylic groups (COO^−^) of glutamate (p*K*_a_ = 4.3), whereas protonated amino groups (NH_3_^+^) of lysine (p*K*_a_ = 10.5) further stabilize the aggregate by cation–π interactions with the aromatic ring of dopamine (Figure 1c). For this, K and E residues must be next to each other. Even a single glycine residue (G) located between K and E can destabilize the aggregates [13]. Among such possible additives, the albumin protein is an interesting candidate since it contains one KE diad and is already widely used in biology. Its hydrodynamic radius of about 4 nm at pH 7 makes it possible to envisage NP sizes close to 10 nm [20]. Chassepot and Ball prepared eumelanin-like particles in the presence of albumin, whose sizes decreased with the amount of protein down to 30 nm in diameter [14]. The structure of these albumin/PDA NPs has not been elucidated completely. It has been demonstrated that proteins are present in the NPs’ shell; they might also be present in the core (Figure 1d). Nevertheless, their potential both for fluorescent labelling of alive bacterial cells and as nanovector for antibacterial activity is high because of their small size and because any antibacterial natural or synthetic peptide containing KE diads may be used to create such PDA NPs. Fluorescent PDA NPs made with a KE diad-containing protein or peptide have never been reported so far. They may be obtained by labelling with a fluorescent dye or oxidizing them, as reported by Ma et al. for other PDA NPs [21].

However, the capacity of PDA-based nanoparticles to target and penetrate bacteria for drug delivery, photothermic action, or fluorescent labelling of cells is unknown. Few publications have reported that nanoplastics can penetrate and accumulate in bacterial cells [22], thus suggesting that other ONPs may have a similar fate in bacteria. In general, the mechanisms of action of ONPs used as drug nanocarriers in antibacterial applications are expected to vary with the nanoparticle type (e.g., liposomes or PLGA NPs) but have not been elucidated so far. In contrast to inorganic NPs [23–24], it is unclear whether ONPs can penetrate bacterial cells. Alipour et al. have shown that a 170 nm diameter liposomal nanocarrier increased the accumulation of an antibiotic (polymyxin B) in Gram-negative bacterial cells, but the penetration of liposomes into the cell was not proved [25]. In general, organic nanocarriers are often reported to penetrate mammalian cells infected by bacteria, improving the drug accumulation in these eukaryotic cells and increasing the antibacterial efficiency of the drug [3–4,9,26]. However, the nanocarriers were not found in the bacterial cells, and the question was rarely mentioned at all. Thus, whether the increase in effectiveness of antibiotics carried by NPs is the result of the penetration of the complete nanocarrier–drug system into bacteria or rather an effect of the destabilization of the bacterial cell membrane by interactions with the nanocarriers (thus allowing for the penetration of the drug into the bacteria) is not known (Figure 1e). Yet, the accumulation of ONPs in bacterial cells is crucial if ONPs are to be used for fluorescent labelling of cells. Also, in the case of nanocarrier–drug systems, it may increase the dose of drug delivered close to the bacterial cell machinery and, therefore, improve the treatment efficacy. If the photothermic properties of the ONPs are to be exploited, the efficacy of the antibacterial treatment may also completely depend on the capacity of ONPs to penetrate and accumulate in the bacterial cell. Therefore, the fate of PDA NPs related to bacterial cells is a crucial aspect for their further use in antibacterial applications.

The objective of the study was to synthesize fluorescent PDA NPs based on a lysine–glutamate-diad containing protein and to determine whether they can enter and accumulate in bacterial cells. The investigation has been conducted with NPs made of polydopamine (PDA) and bovine serum albumin (BSA), and *Escherichia coli* (*E. coli*) bacteria as a bacterial model. Three different types of fluorescent BSA/PDA NPs have been designed to allow for the modification of their fluorescence properties. This also modified the outer surface chemistry; thus, the ability of the NPs to pass through the cell membrane was possibly facilitated. The localization of the fluorescent BSA/PDA NPs related to the cells was investigated by high-resolution fluorescence imaging (high-resolution confocal microscopy). Also, the capacity of the pristine and fluorescent NPs to inhibit the growth of bacterial cells was evaluated through minimal inhibitory concentration (MIC) tests. The MIC value was also determined for *Staphylococcus aureus* (*S. aureus*).

## Results and Discussion

Pristine BSA/PDA NPs with BSA/DA ratios ranging from 0.25 to 10 and three different types of fluorescent BSA/PDA NPs with a BSA/DA ratio of 10 were synthesized and characterized. Three synthesis routes were used to prepare these fluorescent BSA/PDA NPs, based either on the oxidation of pristine BSA/PDA NPs (Ox-BSA/PDA NPs) or on labelling with fluorescein 5-isothiocyanate (FITC) or rhodamine B isothiocyanate (RhBITC) fluorescent dyes (FITC-BSA/PDA NPs and RhBITC-BSA/PDA NPs, respectively). According to Ma et al., Ox-BSA/PDA NPs are expected to emit a maximum of fluorescence in the 450–500 nm range under 405 nm light irradiation [21]. FITC-BSA/PDA NPs and RhBITC-BSA/PDA NPs are expected to emit similarly to free FITC (emission in the green range if excited at 488 nm) and RhBITC (emission in the red range if excited at 561 nm), respectively.

### BSA/PDA NPs can be as small as 10 nm

#### Synthesis of stable BSA/PDA NPs with size control

**Figure 2:**
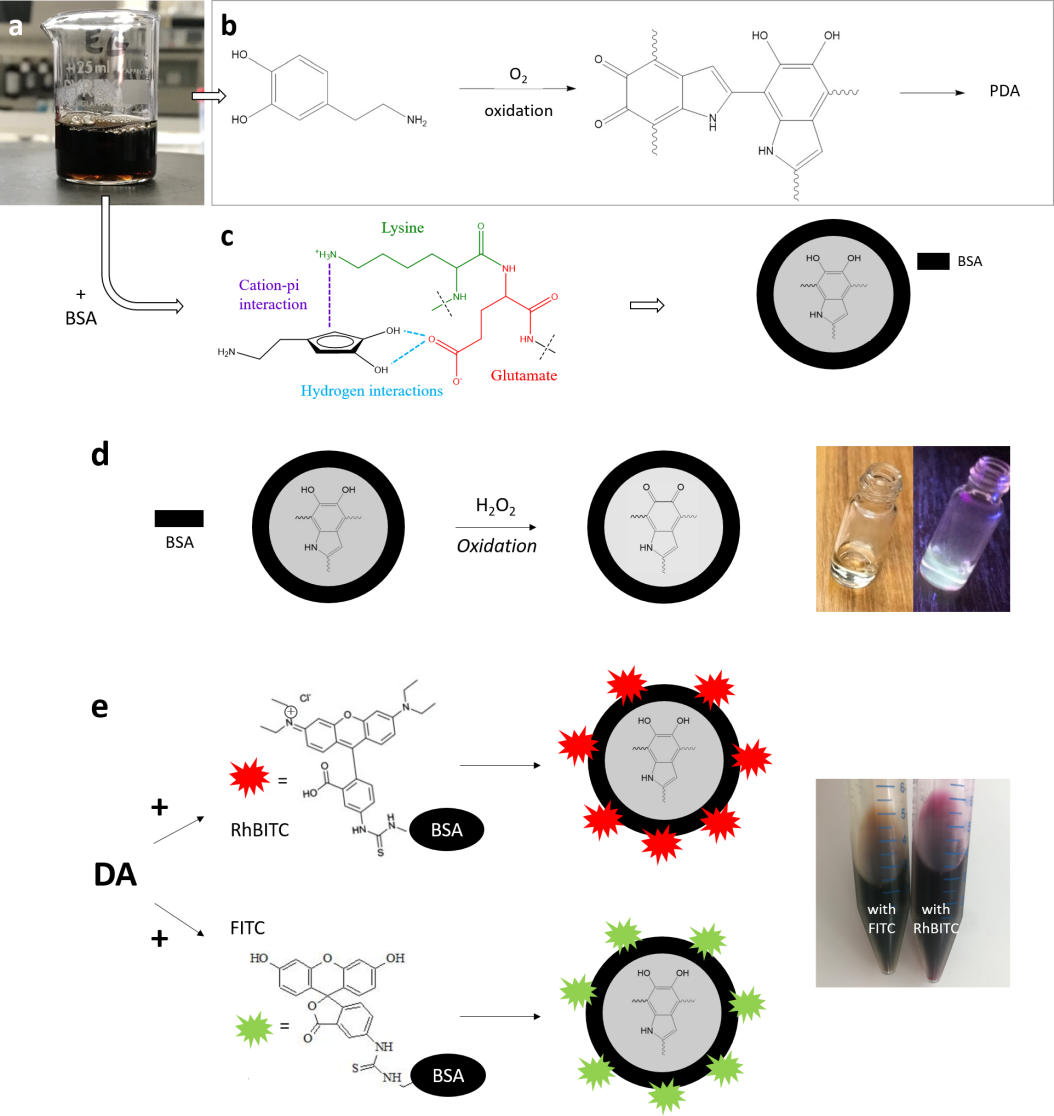
(a) Photographic image of a dopamine solution. Main reactions leading to an auto-polymerization of dopamine to (b) polydopamine or (c) to polydopamine nanoparticles in the presence of BSA (inspired from [13]). (d) Synthesis principle and photographic images of suspensions of fluorescent Ox-BSA/PDA NPs obtained by oxidation of pristine BSA/PDA NPs. (e) Synthesis principles and photographic images of fluorescent RhBITC-BSA/PDA NPs and FITC-BSA/PDA NPs obtained by using RhBITC- and FITC-labelled BSA, respectively.

**Figure 3:**
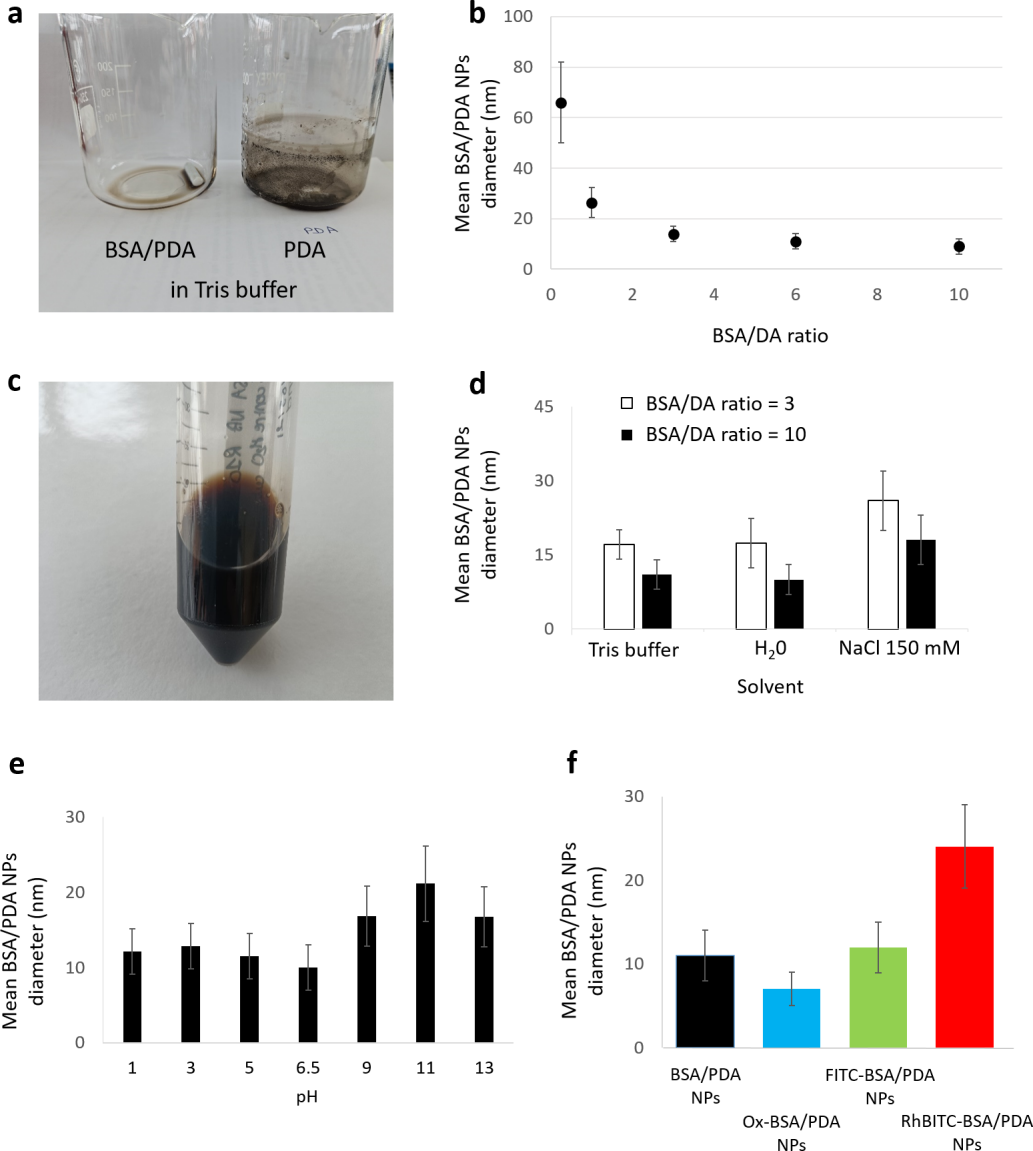
Solutions and sizes of pristine and fluorescent BSA/PDA NPs. (a) Evidence of the inhibition of a PDA film deposition on the wall of the reaction beaker of (left) BSA/PDA solution in Tris buffer compared to (right) PDA solution in Tris buffer. (b) Mean hydrodynamic diameter of pristine BSA/PDA NPs as a function of the BSA/DA ratio. (c) Photograph of pristine BSA/PDA NPs after two years of storage. (d) Mean hydrodynamic diameter in number of pristine PDA/BSA-NPs depending on the solvent. (e) Mean hydrodynamic diameter in number of pristine PDA/BSA-NPs depending on the pH value. (f) Mean hydrodynamic diameter in number of pristine BSA/PDA NPs, Ox-BSA/PDA NPs, FITC-BSA/PDA NPs, and RhBITC-BSA/PDA NPs (all synthesized with a BSA/DA ratio of 10).

Pristine polydopamine nanoparticles (BSA/PDA NPs) were prepared according to Bergtold et al. [13] (Figure 2a–c). BSA and DA solutions were mixed in Tris buffer with ratios varying from 0.25 to 10. Contrary to a DA solution in Tris buffer, the mixtures of BSA and DA in Tris buffer inhibited the deposition of a PDA film on the wall of the reaction beakers (Figure 3a). This suggested that BSA/PDA aggregates formed and that almost all the free DA molecules were consumed in these aggregates, as already mentioned by Bergtold and co-workers [13].

A series of pristine BSA/PDA NPs with a mean diameter in number ranging from 66 ± 16 to 9 ± 3 nm was obtained (Figure 3b; see Supporting Information File 1, Table S1 for mean diameter in intensity) by increasing BSA/DA ratio from 0.25 to 10. The formation of BSA/PDA NPs is attributed to the interaction between DA and BSA, which contains the KE diad and allows for the control of the NP formation [13]. During the synthesis, DA is added into the BSA solution, thus avoiding that the proteins come into contact with already formed PDA. Bergtold et al. proposed that the size control is exerted by the specific interaction of the KE diad with DA [13]. Most of the DA molecules interact with the proteins, thus limiting the binding of proteins with each other or with PDA aggregates. This results in an increase in aggregate (i.e., particle) size. Hence, a rapid decrease in diameter was observed for BSA/DA ratios from 0.25 to 3. However, a plateau value was reached above a ratio of 6, leading to BSA/PDA NPs of 11 ± 3 nm and 9 ± 3 nm in diameter for BSA/DA ratios of 6 and 10, respectively. The size of the BSA protein is probably the limiting factor to a further decrease of the NP size. Indeed, one BSA molecule has an hydrodynamic diameter of 3–4 nm at physiological pH [20].

The resulting number *N* of nanoparticles per milliliter and self-polymerization reaction yield η obtained with BSA/DA ratios of 3 and 10 were calculated with Equation 1 and Equation 2, respectively:


[1] $$N=\frac{m_{\text{dry}}}{V}\times \frac{1}{\frac{4}{3}\pi r^{3}\rho_{\text{PDA}}},$$

where *V* is the volume of the NP solution remainder after freeze-drying, *m*_dry_ is the mass of the NP foam measured after freeze-drying, and ρ_PDA_ is the density of polydopamine (1.52 g·cm^−3^) [27].


[2] $$\eta =\frac{C_{\text{f}}}{C_{i}^{\text{DA}}+C_{i}^{\text{BSA}}},$$

where *C*_f_ is the final concentration after freeze-drying (*m*_dry_/*V*), 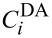 is the initial concentration of dopamine (2 mg/mL), and 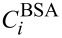 is the initial concentration of BSA (6 mg/mL and 20 mg/mL for BSA/DA ratios of 3 and 10, respectively).

Final concentration and number of BSA/PDA NPs increased by increasing the BSA/DA ratio from 3 and 10 (2 × 10^14^ and 4 × 10^15^ NPs/mL, respectively), indicating a similar reaction yield of 85% (Supporting Information File 1, Table S2). The dispersion was changed by changing the ratio of BSA, but the reaction yield was maintained. This suggests that BSA acted as a “knife” changing the size, but not the composition, of the nanoparticles.

The suspension of BSA/PDA NPs was stable over two years of storage in Tris buffer in the dark at ambient temperature, as shown by the good dispersion and the absence of precipitates and deposition on the container wall (Figure 3c). Good stability and dispersion were maintained after dialysis with a 100 kDa cut-off membrane, which allowed for the removal of free BSA molecules. As shown by Chassepot and Ball [14] and Bergtold et al. [13], human serum albumin and other proteins play an important role in size control and stability of PDA NPs. Similarly, the stability of BSA/PDA NPs is probably due to BSA thanks to the strong PDA/KE interactions reported by Bergtold et al. This is supported by the progressive inhibition of the deposition of a PDA film on the wall of the reaction beaker, which was mentioned above and observed above a certain amount of BSA (Figure 3a). This allowed us to assume that BSA molecules below a critical amount cannot surround all DA molecules (some DA molecules thus form a thin film of PDA on the beaker’s wall), whereas they stabilize PDA NPs above this amount.

The suspension stability was maintained when BSA/PDA NPs were in suspension in H_2_O or 150 mM NaCl solution. However, the mean hydrodynamic diameter varied with the solvent, with an increase by up to 50% for a BSA/DA ratio of 3 and 60% for a BSA/DA ratio of 10. The smallest BSA/PDA NPs had a mean diameter of 9 nm in 50 mM Tris and of 10 nm in H_2_O, but of 18 nm in 150 mM NaCl solution (Figure 3d). This increase was attributed to two co-existing phenomena related to the high salt concentration in 150 mM NaCl. First, the high ionic strength of 150 mM NaCl decreased long-range effect and intensity of electrostatic interactions compared to the other solvents, thus favoring other types of interactions (such as attractive van der Waals interactions) and, therefore, aggregation. Second, the increase in salt concentration might have led to an increase in the Stern layer thickness, resulting in a larger hydrodynamic diameter measured by DLS.

The size of BSA/PDA NPs remained stable as a function of pH at acidic pH, but increased moderately under alkaline conditions (Figure 3e). Chen et al. reported that PDA NPs deteriorated above pH 11 with, first, a decrease in size before the NP morphology changed into nanosheets [28]. However, in the case of BSA/PDA NPs, the NPs size increased when the pH value was above 6.5. In addition, precipitation, sedimentation, or degradation of the dispersion were not observed, showing that the suspension stability was unchanged. These results suggest that BSA prevented the degradation in alkaline solution, probably through heavily stabilizing the NPs.

The BSA/PDA NPs suspensions were freeze-dried to obtain dried NPs that could easily be stored and re-suspended at the needed concentration for further experiments. During the freeze-drying process, BSA/PDA NPs acted as surfactant and were located around water droplets when the water was rapidly sublimated, which resulted in the formation of a foam (Supporting Information File 1, Figure S1a). After freeze-drying, the foam could be easily re-suspended in Milli-Q^®^ water, resulting in a well-dispersed black suspension (Supporting Information File 1, Figure S1b). However, in the case of a BSA/DA ratio of 0.25, a powder was obtained instead of a foam (Supporting Information File 1, Figure S1c). This was probably due to a lack of BSA molecules to surround every molecule of PDA, which may have favored bridges involving BSA between NPs and further formation of aggregates (Supporting Information File 1, Figure S1d). This powder could not be re-dissolved in water, NaCl, or Tris buffer. Furthermore, the mean hydrodynamic diameter slightly increased for both BSA/DA ratios of 3 and 10 after dialysis and freeze-drying (22% and 18%, respectively) (Supporting Information File 1, Figure S1e). During water evaporation, NPs may have been brought closer, leading to the formation of irreversible interactions and slight aggregation. This only slight increase in size was considered insignificant regarding the possible effect on the interactions of NPs with bacteria; hence, freeze-drying was further used for the storage of BSA/PDA NPs.

#### Fluorescent modifications of BSA/PDA NPs

BSA/PDA NPs were modified to produce fluorescent BSA/PDA NPs by three different methods, that is, the oxidation of pristine BSA/PDA NPs (Ox-BSA/PDA NPs) (Figure 2d) or the labelling of BSA with FITC or RhBITC fluorescent dyes (FITC-BSA/PDA NPs and RhBITC-BSA/PDA NPs, respectively) (Figure 2e). All fluorescent BSA/PDA NPs were made from pristine BSA/PDA NPs synthesized with a BSA/DA ratio of 10. This resulted in Ox-BSA/PDA NPs and FITC-BSA/PDA NPs with a mean diameter similar to that of pristine BSA/PDA NPs and RhBITC-BSA/PDA NPs of about 20 nm in diameter (Figure 3f). A small decrease in size of Ox-BSA/PDA NPs was expected and measured (30% decrease), as also noticed by Ma et al., probably due to the degradation of the pristine BSA/PDA NPs upon oxidation [21]. However, regarding the size values and deviations measured, the decrease was considered insignificant here. The increase in size of RhBITC-BSA/PDA NPs upon labelling with RhBITC can be explained by the simultaneous presence of a positive charge and two additional diethylamino groups in RhBITC, which are absent in FITC. These two additional chemical moieties may have induced some weak aggregation between the negatively charged RhBITC-BSA/PDA NPs, in contrast to FITC-BSA/PDA NPs.

Ox-BSA/PDA NPs were produced by the oxidation of BSA/PDA NPs for 24 h with H_2_O_2_. The BSA/PDA NP suspension, initially dark brown, turned to translucent light brown during oxidation (Supporting Information File 1, Figure S2b). Under UV light, the Ox-BSA/PDA NPs suspension emitted fluorescence, in contrast to the suspension before oxidation (Supporting Information File 1, Figure S2c). This may result from the conjugation and electronic density changes induced during the reaction with H_2_O_2_, during which hydroxy groups were oxidized into ketones. Eventually, the large majority of polydopamine functional groups are expected to be ketones with only a few remaining of hydroxy groups (Supporting Information File 1, Figure S2a). In addition, labelling of pristine BSA/PDA NPs with FITC and RhBITC dyes led to an obvious change in color of the suspension, from brown (Supporting Information File 1, Figure S3a) to yellow and red (Supporting Information File 1, Figure S3b,c), respectively, in agreement with the color of the corresponding dye.

**Figure 4:**
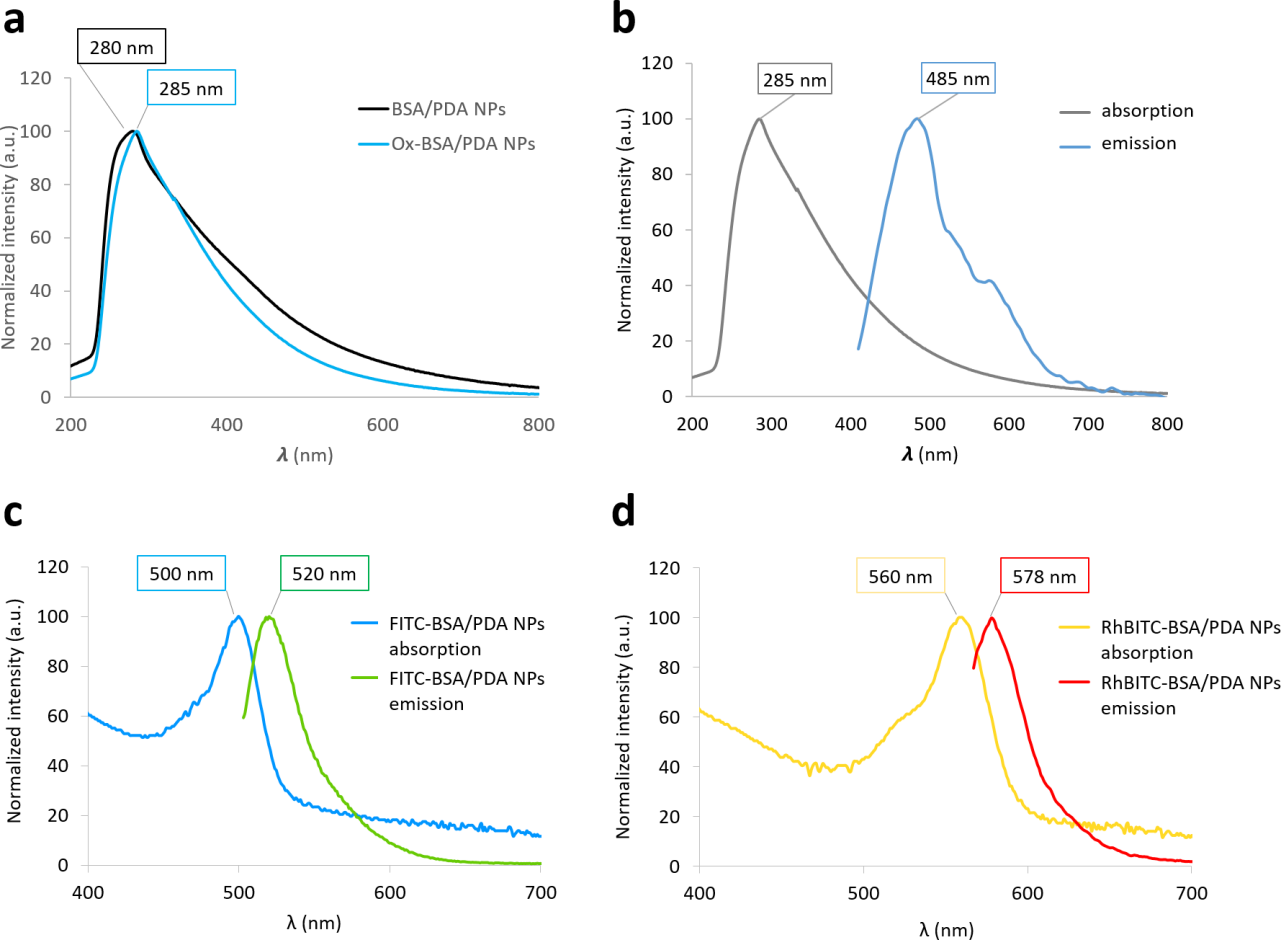
Absorption and emission spectra of pristine BSA/PDA NPs, Ox-BSA/PDA NPs, FITC-BSA/PDA NPs, and RhBITC-BSA/PDA NPs. (a) Absorption of pristine BSA/PDA NPs before and after oxidation. (b) Normalized absorption and fluorescence emission (excitation wavelength λ_exc_ = 375 nm) spectra of Ox-BSA/PDA NPs. (c) Normalized absorption and fluorescence emission (excitation wavelength λ_exc_ = 488 nm) spectra of FITC-BSA/PDA NPs. (d) Normalized absorption and fluorescence emission (excitation wavelength λ_exc_ = 550 nm) spectra of RhBITC-BSA/PDA NPs. Spectra were smoothed using the moving average method on three points.

Pristine BSA/PDA-NPs exhibited a wide absorption spectrum with a maximum of absorption at 280 nm, whereas Ox-PDA/BSA-NPs showed a less broad and intense absorption with a maximum at 285 nm (Figure 4a). In other words, the oxidation leads to a bathochromic (increase in wavelength) shift due to an increase in conjugation when –OH groups were oxidized into =O groups. Moreover, a hypochromic (decrease in absorption intensity) effect was measured, which may have resulted from the degradation of the BSA/PDA-NPs upon oxidation (suggested by the insignificant decrease in size of Ox-BSA/PDA NPs compared to pristine BSA/PDA NPs) and a reduction of concentration or molar extinction coefficient during the prolonged oxidation. Indeed, it is well known that the oxidation of indole quinone groups in melanin-like materials leads to the release of pyrrole carboxylic acids [29]. In addition, Ox-BSA/PDA NPs revealed an emission spectrum centered around 490 nm. It is to note that no significant shift of the maximum emission wavelength depending on the excitation wavelength was measured (Supporting Information File 1, Figure S4a), contrary to what has been mentioned in the literature [21,30]. This effect is not elucidated so far, but it is important to note that the NPs considered here are different from those of Zhang and Ma, since PDA is here associated to BSA. Moreover, the Stokes shift of Ox-BSA/PDA NPs (λ_max ems_ − λ_max abs_) is estimated to be about 200 nm (Supporting Information File 1, Figure S4b), which is higher than that of common fluorochromes such as RhBITC and FITC (about 25 nm) [31] or even DAPI (about 100 nm) [32]. This big difference in energy is consistent with the ability of PDA to heat up under irradiation by non-radiative relaxation. This is favorable to minimize the superposition between absorption and emission peaks, but might lead to a low quantum yield and, thus, poor emission intensity. Indeed, the quantum yield of Ox-BSA/PDA NPs in water (Φ) was calculated to be 0.1% with Equation 3, using DAPI in water as the reference [33].


[3] $$\begin{array}{c} \Phi =\Phi_{\text{DAPI}}\times \frac{I_{\text{fluo}}^{\text{Ox-BSA/PDA NPs}}}{I_{\text{fluo}}^{\text{DAPI}}}\\ \times \frac{A_{\text{DAPI}}}{A_{\text{Ox-BSA/PDA NPs}}}\times \frac{\eta_{\text{Ox-BSA/PDA NPs}}^{2}}{\eta_{\text{DAPI}}^{\text{2}}}, \end{array}$$

where, Φ_DAPI_ is the fluorescence quantum yield of DAPI when unbound to DNA (4%) [32], 
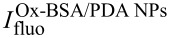
 and 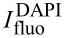 are the integrated fluorescence intensities of the light emitted by Ox-BSA/PDA NPs and DAPI, respectively, *A*_Ox-BSA/PDA NPs_ and *A*_DAPI_ are the absorption values of Ox-BSA/PDA NPs and DAPI solutions, respectively, and η_Ox-BSA/PDA NPs_ and η_DAPI_ are the refractive indices of the solvents of Ox-BSA/PDA NPs and DAPI solutions, respectively. Because the solvent is the same (water) for both Ox-BSA/PDA NPs and DAPI solutions, the value of 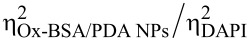 is here equal to 1.

Compared to the quantum yields of FITC, RhBITC, and DAPI bound to DNA, which are in the range of 40–90% at room temperature [31–32,34], Ox-BSA/PDA NPs reveal low emission efficiency. However, even if the quantum yield of Ox-BSA/PDA NPs is low, it is significantly higher than the quantum yield of non-oxidized NPs, which are not fluorescent at all [35].

FITC- and RhBITC-BSA/PDA NPs exhibited absorption maxima at 500 and 560 nm, respectively, shifted by 8 nm compared with free FITC and RhBITC (absorption maxima at 492 and 552 nm, respectively) (Figure 4c,d and Supporting Information File 1, Figure S5). This probably results from a change in polarity in the close environment of the bound fluorophore. In addition, the absorption intensity of FITC-BSA/PDA and RhBITC-BSA/PDA NPs above 600 nm is a typical feature of melanin materials. The high background (decreasing from 400 to 800 nm and similar to the absorption spectrum of pristine BSA/PDA NPs) was attributed to the absorption by NPs (Figure 4a). The emission spectra of FITC- and RhBITC-BSA/PDA NPs were centered around 520 and 578 nm, respectively, which is similar to free FITC and RhBITC (emission maxima at 519 and 578 nm, respectively) under excitation at 488 and 550 nm, respectively (Figure 4c,d and Supporting Information File 1, Figure S5). Unfortunately, because PDA absorbs a significant part of the light emitted by the fluorophores, the intensity cannot be related to the number of FITC- and RhBITC-labelled NPs. However, the absorption intensities were used to estimate (even though roughly) the equivalent quantities of free FITC and RhBITC to be used as controls in the further bacteriological assays. The estimated equivalent quantities corresponding to a concentration of fluorescent NPs of 2 mg/mL (164 µM for FITC and 373 µM for RhBITC) were calculated as described in the Experimental section.

The stability of the fluorescent labelling was tested after four months of ageing of FITC- and RhBITC-BSA/PDA NPs. Dialysis was performed on the four months old NP suspensions to retrieve FITC or RhBITC molecules possibly released from the labelled NPs by following the same procedure as described below for the elimination of unbound labelling molecules (see Experimental section). The fraction of released dye was estimated on the basis of the emission intensity of the dialysate and the initial quantity of labelled NPs (Supporting Information File 1, Figure S6). The fractions were less than 1% (1.5 µM for FITC and 0.5 µM of RhBITC) of the initial dye quantity, showing the high stability of the labelling and of the labelled NPs.

#### FITC-BSA/PDA NPs and RhBITC-BSA/PDA NPs tend to accumulate in *E. coli* cells with heterogeneous patterns

Whether pristine and fluorescent BSA/PDA NPs can interact with bacterial cells (especially *E. coli*) is unknown. Therefore, the possible accumulation of pristine and fluorescent BSA/PDA NPs on and in cells was studied with the smallest NPs (about 10 nm diameter) since NP penetration in cells is expected to be inversely related to NP size [36].

The accumulation of pristine and fluorescent BSA/PDA NPs was evaluated by standard and high-resolution fluorescence confocal microscopy after 24 h of contact of NPs with *E. coli* cells. Obviously, bacteria without NPs and bacteria with pristine BSA/PDA NPs were not detected at excitation and emission wavelengths corresponding to FITC and RhBITC dyes. It was also impossible to identify the specific fluorescent signal emitted by Ox-BSA/PDA NPs in absence of an adequate laser for the excitation of Ox-BSA/PDA NPs at 285 nm. Under excitation with a higher wavelength (405 nm), a low fluorescence signal was detected in the 460–541 nm and 415–482 nm ranges with the standard and the high-resolution microscope, respectively (Supporting Information File 1, Figure S7a). In part, it may result from the fluorescence of Ox-BSA/PDA NPs at λ_exc_ = 405 nm; however, it is more probably due to the intrinsic fluorescence of bacteria. Indeed, *E. coli* cells alone and *E. coli* cells cultivated with free FITC or RhBITC, or FITC- or RhBITC-BSA/PDA NPs revealed a similar fluorescence signal when excited at 405 nm (Supporting Information File 1, Figure S7a). This can be attributed to the intrinsic fluorescence of bacteria under 405 nm excitation due to porphyrins [37–38]. With Ox-BSA/PDA NPs, no significant difference in fluorescence intensity could be detected in this emission range under 405 nm excitation compared to the intrinsic fluorescence background. This prevents a conclusion on whether Ox-BSA/PDA NPs may have accumulated on or in *E. coli* cells.

**Figure 5:**
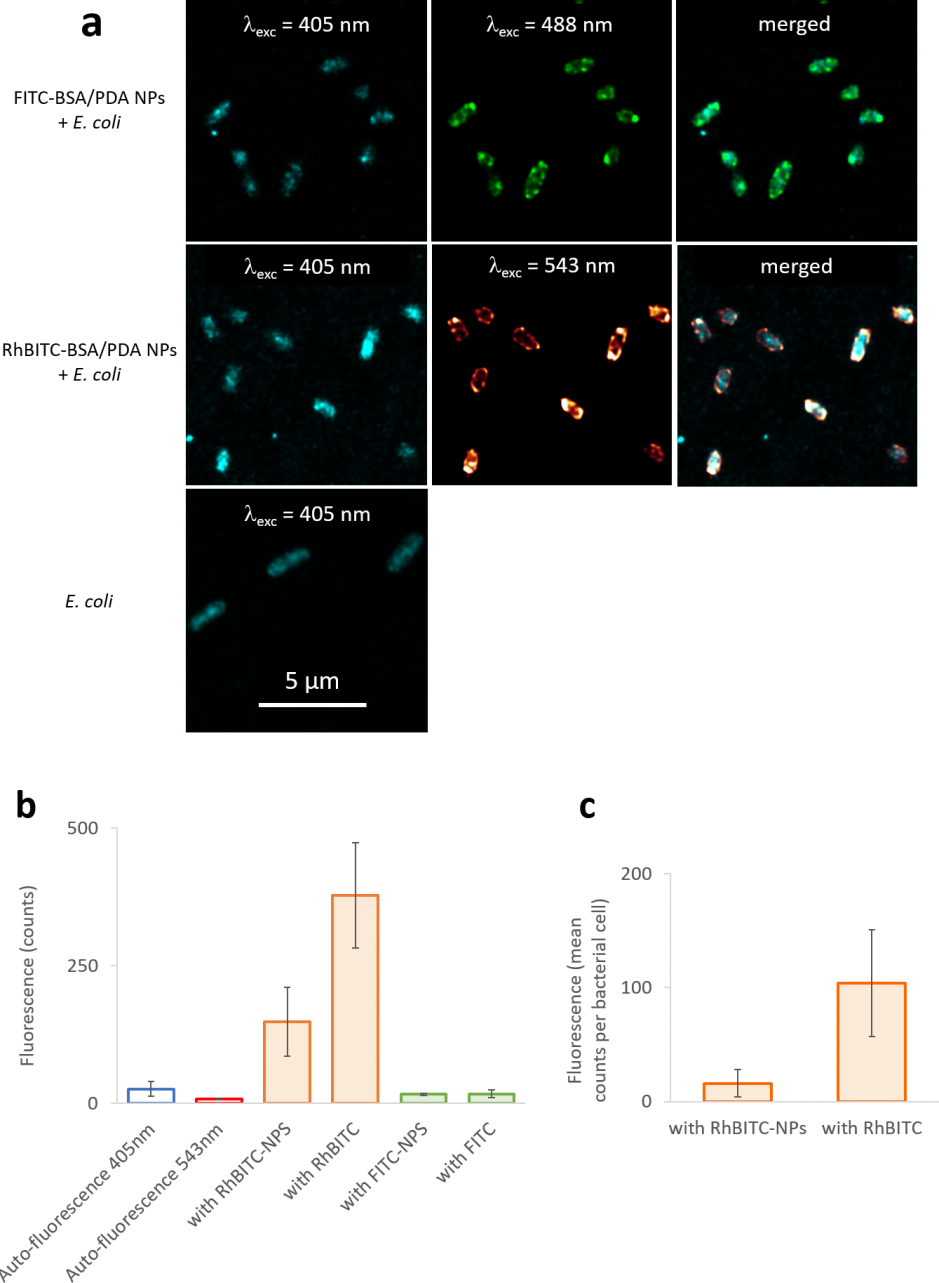
Fluorescence characterization of *E. coli* cultures with FITC-BSA/PDA NPs, free FITC, RhBITC-BSA/PDA NPs, free RhBITC, or alone (NPs synthesized with a BSA/DA ratio of 10). (a) Typical fluorescence micrographs extracted from 3D-stack images measured with a high-resolution CLSM (Stellaris 5, Leica Biosystems, Wetzlar, Germany) (λ_exc_ of 405, 488, or 543 nm; range of λ_em_ of 415–482, 496–565, and 553–628 nm, respectively). (b) Maximal fluorescence intensity measured in bacterial cells for each condition (mean ± SD of all the micrographs of each condition). (c) Mean fluorescence intensity of bacterial cells for each condition (mean counts in bacterial cells per field) (mean ± SD of all complete bacteria in all micrographs of each condition).

Under excitation at 488 nm, cells cultivated with FITC-BSA/PDA NPs and free FITC emitted slight fluorescence in the 495–634 nm and 496–565 nm ranges (observed with standard and high-resolution microscopes, respectively) (Figure 5a, Supporting Information File 1, Figure S7c), in contrast to *E. coli* cells alone or cultivated with RhBITC-BSA/PDA NPs or free RhBITC. Under excitation at 561 nm, *E. coli* cells cultivated with RhBITC-BSA/PDA NPs or free RhBITC emitted fluorescence in the 567–703 nm and 553–628 nm ranges (observed with standard and high-resolution microscopes, respectively) (Figure 5a, Supporting Information File 1, Figure S7). These emission ranges are in agreement with the fluorescence characteristics of FITC-BSA/PDA NPs and RhBITC-BSA/PDA NPs (Figure 4c,d) and can, thus, be attributed to FITC or RhBITC. Hence, FITC alone, RhBITC alone, FITC-BSA/PDA NPs, and RhBITC-BSA/PDA NPs were all able to label *E. coli* cells. However, a much higher intensity was observed with RhBITC and RhBITC-BSA/PDA NPs than in the other experiments as confirmed by quantifying the maximal fluorescence measured in the micrographs (Figure 5b). This may result from the positive charges carried by RhBITC at physiological pH, which are absent in the FITC molecules. The positive charges are expected to participate in attractive electrostatic interactions with the negatively charged bacterial membrane, that is, the phosphate groups of their phospholipids. This facilitates the intimate contact between RhBITC-BSA/PDA NPs and the bacterial membrane and, possibly, the subsequent penetration of the NPs.

**Figure 6:**
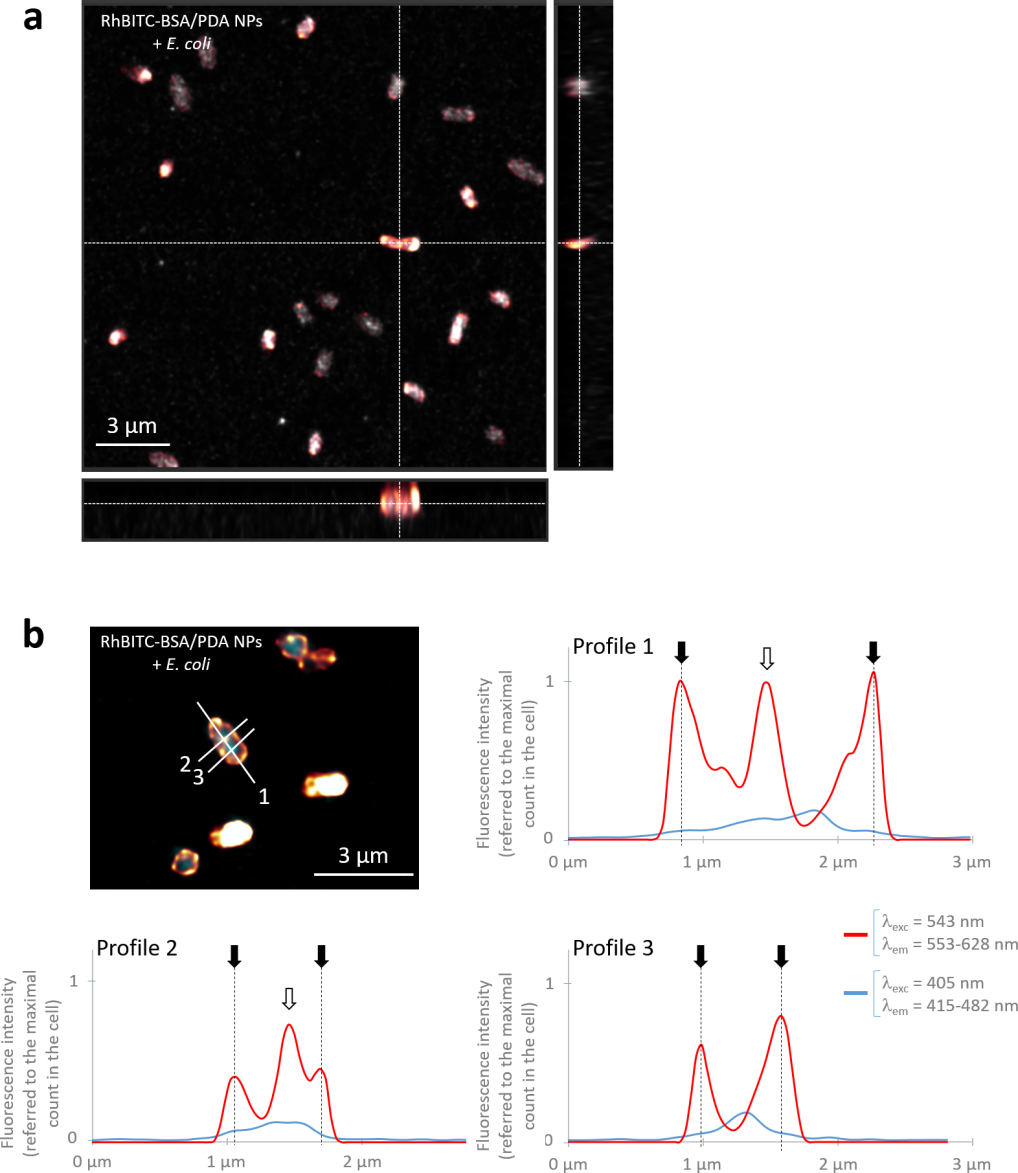
Localization of the fluorescence emission related to *E. coli* cells. (a) Orthogonal views extracted from a 3D stack of a RhBITC-BSA/PDA NPs with *E. coli* sample measured with a high-resolution CLSM (Stellaris 5, Leica Biosystems, Wetzlar, Germany) (λ_exc_ of 405 and 543 nm; range of λ_em_ of 415–482 and 553–628 nm, respectively). (b) Longitudinal and transversal profiles of fluorescence intensity of a typical bacterial cell cultivated with RhBITC-BSA/PDA NPs (profiles are identified with numbers as displayed on the micrograph measured with the high-resolution Stellaris 5 CLSM as described in (a)).

The localization of the fluorescence emission related to bacterial cells was determined on the basis of micrographs extracted from 3D-stack images obtained with the high-resolution confocal laser scanning microscope (CLSM, Stellaris 5, Leica Biosystems, Wetzlar, Germany). In all experiments with dyes (FITC-BSA/PDA NPs, RhBITC-BSA/PDA NPs, free FITC, and free RhBITC in equivalent concentrations), the fluorescence signal related to RhBITC or FITC was emitted at the location of the cells (Figure 5a,c and Figure 6). The fluorescence intensity in cells incubated with RhBITC-BSA/PDA NPs was about 15% of the intensity in cells incubated with the equivalent quantity of free RhBITC (Figure 5b). It reached about 100% with FITC-BSA/PDA NPs compared to free FITC. These ratios are much higher than expected if only free dye molecules released from NPs had penetrated the cells (1% as estimated above). Hence, they prove that the fluorescence measured in cells was not emitted by free molecules of dye but by molecules bound to NPs.

More specifically, the fluorescence appeared to come from the cell wall and from some inner parts of the bacterial cells with a frequent location at the poles of the cell. The fluorescence profiles confirm (i) that dyes accumulate in the cell wall rather than on the outside as shown by the co-localization of the intrinsic (blue) and dye-related fluorescence signals (dashed lines and black arrows in Figure 6b, Supporting Information File 1, Figure S8) and (ii) that the fluorescence was present as clusters in inner cell compartments (empty arrows in Figure 6b, Supporting Information File 1, Figure S8). This accumulation of labelled BSA/PDA NPs may be related to proteins since fluorescent dyes such as RhBITC can interact with proteins in a non-covalent manner [39]. In addition, proteins can aggregate at the poles or in the cytosol of *E. coli* (and other bacterial species) during cell division or as the result of cellular ageing or under external stressors [40–41]. This may match the distribution patterns observed with FITC- and RhBITC-BSA/PDA NPs. Moreover, by themselves or by the excitation light used to reveal them, the labelled NPs may be the external stressor leading to cell ageing and subsequent protein aggregation as proposed by Rang and co-workers [42]. In any case, our results indicate that FITC- and RhBITC-BSA/PDA NPs were able to penetrate and accumulate in the cell wall and internal compartments of *E. coli* cells in significant quantities.

**Figure 7:**
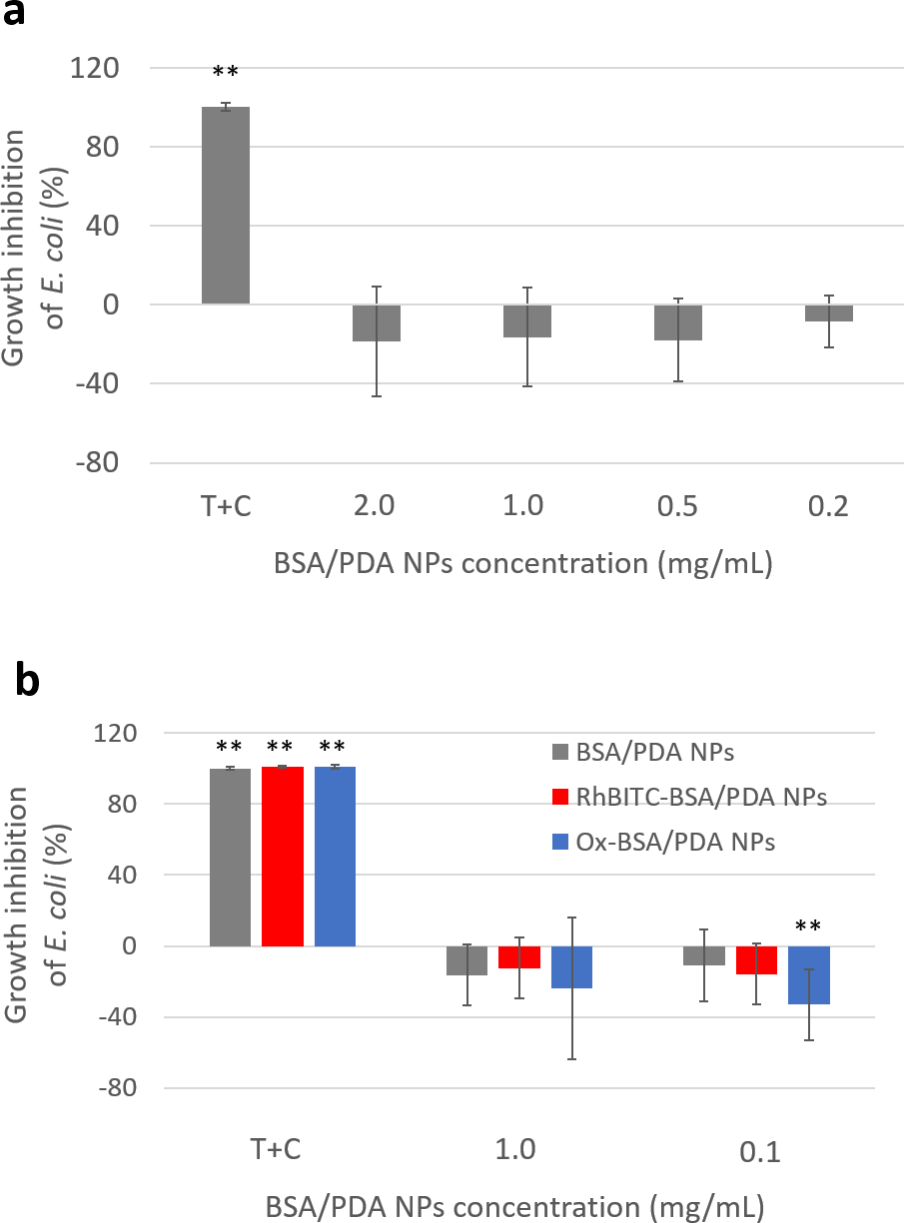
Growth inhibition of *E. coli* populations with pristine BSA/PDA NPs from 0.2 to 2 mg/mL concentration or with antibiotics (solution of 10 µg/mL tetracycline and 0.1 µg/mL cefotaxime) (“T+C”; positive control) compared to *E. coli* culture without NPs (a), and with pristine and fluorescent Ox-BSA/PDA NPs and RhBITC-BSA/PDA NPs (1 mg/mL concentration) compared to *E. coli* culture without NPs (b). * and ** indicate significant differences to *E. coli* culture alone (*p* < 0.01 and *p* < 0.001, respectively).

BSA/PDA NPs are prone to damage bacterial cells since they can penetrate them. The possible harmful impact on bacterial growth was therefore tested with *E. coli* and *S. aureus*. However, we showed that BSA/PDA NPs in concentrations corresponding to more than 10^7^ NPs per bacterial cell (0.2–2.0 mg/mL; 4 × 10^13^ to 4 × 10^14^ NPs/mL) were unable to significantly modify the growth of a *E. coli* population (Figure 6a). This was maintained regardless of the surface properties of the NPs since pristine, Ox-, and RhBITC-BSA/PDA NPs all failed to inhibit *E. coli* growth (Figure 7b). It can be noted that the population growth was even slightly but significantly favored in the presence of pristine and fluorescent BSA/PDA NPs. This effect, which is not elucidated so far, was also observed regarding the growth of *S. aureus* populations (Supporting Information File 1, Figure S9). Especially the absence of *E. coli* growth inhibition by RhBITC-BSA/PDA NPs allows us to envisage the use of the labelled BSA/PDA NPs developed in this study to track bacterial cells, but also to carry drugs in bacterial cells thanks to their penetration capacity.

## Conclusion

Fluorescent nanoparticles of PDA with about 15 nm diameter were synthesized with BSA as a KE diad-carrying protein. Blue fluorescence was obtained under 405 nm excitation by oxidation of pristine BSA/PDA NPs, while green fluorescence under 488 nm illumination and red fluorescence under 543 nm illumination were achieved by labelling pristine BSA/PDA NPs with fluorescent FITC and RhBITC dyes, respectively. The fluorescent NPs did not significantly vary in size compared to pristine NPs, with diameters in the range of 10–15 nm for Ox-BSA/PDA NPs and FITC-BSA/PDA NPs and a slightly higher diameter for RhBITC-BSA/PDA NPs (ca. 20 nm). The suspensions of pristine and fluorescent NPs were all stable over months, allowing for long-term storage. The capacity of FITC- and RhBITC-BSA/PDA NPs to penetrate bacterial cell walls, to enter the cell core, and to accumulate in the different compartments was demonstrated for *E. coli* cells. Both FITC- and RhBITC-BSA/PDA NPs, as well as free FITC and RhBITC dyes, exhibited fluorescence in aggregate-like patterns in the cells. However, the growth of bacteria was not inhibited, which allows us to envisage fluorescent tracking of live bacterial cells using these fluorescent NPs. The capacity of these NPs to penetrate cell walls might also enable their use to carry drugs in the core of bacterial cells.

## Experimental

### Synthesis

#### Reagents

Dopamine (DA) hydrochloride and bovine serum albumin (BSA) were purchased from Merck (Darmstadt, Germany) and used without purification. Fluorescein isothiocyanate (FITC), rhodamine B isothiocyanate (RhBITC), hydrochloric acid 37% (wt/v), absolute ethanol and Hellmanex^®^ were purchased from Merck (Darmstadt, Germany). Tris(hydroxymethyl)aminomethane (Tris buffer) was purchased from Euromedex (Strasbourg, France). 4′,6-Diamidino-2-phenylindole dihydrochloride (DAPI) was purchased from Fisher Scientific (Illkirch, France). All aqueous solutions were made using Milli-Q^®^ water (ρ = 18.2 MΩ·cm; Millipore^®^ Reverse Osmosis system; Merck, Darmstadt, Germany).

#### BSA/polydopamine nanoparticles

Polydopamine nanoparticles (BSA/PDA NPs) were prepared according to Bergtold and co-workers [13] (Figure 2a–c). Tris buffer solution was prepared by dissolution of 6.055 g (5 × 10^−2^ mol) of Tris powder in 1 L of Milli-Q^®^ water. The pH was adjusted to 8.5 by adding hydrochloric acid. *X* mg of BSA (*X* depending on the chosen BSA/DA wt/wt ratio; see Table 1) were slowly dissolved in 100 mL Tris buffer to avoid foam formation. 200 mg of dopamine hydrochloride was added to yield a solution of 2 mg/mL of DA. This solution was gently shaken at ambient temperature in the dark. After 24 h, a homogenous black suspension of BSA/PDA NPs was obtained. Small oligomers, unreacted dopamine monomers, and Tris salt were removed from the reaction medium by dialysis. 20 mL of the suspension were dialyzed against 2 L of Milli-Q^®^ water using a Spectra/Por^®^ dialysis membrane with a molecular weight cut-off of 8–10 kDa (purchased from Fisher Scientific S.A.S., Illkirch, France). The dialysis was performed in the dark and followed by spectroscopic measurements at 280 nm (absorption of dopamine) to check for the absence of oligomers in the dialysate at the end of the dialysis process [13]. Dialysis was repeated three times, yielding a total dilution of the dialysate by a factor of 10^6^. The solution was frozen at −80 °C for 1 h, and water was eliminated by freeze-drying (Alpha 1-4 LD_plus_; Christ, Osterode am Harz, Germany) for 48 h to provide lyophilized foam, which was stored at room temperature and protected from light before use.

**Table 1:** Weight of BSA (*X* mg) and dopamine (DA), and the corresponding BSA/DA ratio used to synthesize the BSA/PDA NPs.

BSA/DA ratio	BSA weight (mg)	DA weight (mg)

0	0	200
0.25	50	200
1.00	200	200
3.00	600	200
6.00	1 200	200
10.00	2 000	200

#### Fluorescent BSA/polydopamine nanoparticles

Fluorescent BSA/PDA nanoparticles with a BSA/DA ratio of 10 were synthesized by using two different ways: (i) by oxidation of pristine BSA/PDA nanoparticles and (ii) by adding fluorescent labels to the BSA used to prepare BSA/PDA nanoparticles (Figure 2d–e).

**PDA oxidation.** Fluorescent, oxidized polydopamine nanoparticles (Ox-PDA/BSA NPs) were prepared on the basis of pristine BSA/PDA NPs synthesized as described above with a BSA/DA ratio of 10 (1.1 × 10^−6^ mol BSA, 1 equiv). Pristine BSA/PDA NPs were then modified by a procedure inspired from Ma et al. [21] as follows: A suspension of BSA/PDA NPs foam was prepared in Milli-Q^®^ water at a concentration of 10 mg BSA/PDA NPs per milliliter; 7.5 mL of this suspension (pH 6.8) and 2 mL of H_2_O_2_ solution (30 wt %) were stirred at room temperature in the dark for 24 h. Residual H_2_O_2_ was removed from the reaction medium by dialyzing about 8 mL of the suspension against 1 L Milli-Q^®^ water using a Spectra/Por^®^ dialysis membrane with a molecular weight cut-off at 8–10 kDa. The dialysis was done in the dark and repeated three times yielding a total dilution of the dialysate by a factor of 10^6^.

**Labelling of BSA/PDA NPs with a fluorescent probe.** Fluorescently labelled polydopamine nanoparticles (FITC- and RhBITC-PDA/BSA NPs) were prepared on the basis of pristine BSA/PDA NPs synthesized as described above with a BSA/DA ratio of 10 (1.1 × 10^−6^ mol BSA, 1 equiv). Pristine BSA/PDA NPs were then modified by labelling BSA with the commercial fluorescent probes FITC and RhBITC to obtain FITC-BSA/PDA NPs and RhBITC-BSA/PDA NPs, respectively. BSA and the fluorescent probes were linked through the isothiocyanate groups of FITC and RhBITC and the amine group of BSA. To prepare FITC-BSA/PDA NPs, 80 mg of dehydrated foam of pristine BSA/PDA NPs were dissolved into 8 mL of sodium carbonate buffer (50 mM, pH 8.5). A concentrated solution of FITC (8.25 mM) was prepared by dissolving 3.2 mg into 1 mL of DMSO. 400 μL of this solution was added to the pristine BSA/PDA NPs solution to reach a FITC concentration of 4 × 10^−4^ M (3.3 × 10^−6^ mol, 3 equiv). The reaction was performed in the dark for 1 h and followed by a dialysis against 1 L of Milli-Q^®^ water using a Spectra/Por^®^ dialysis membrane with a molecular weight cut-off at 8–10 kDa to eliminate unbound labelling molecules. The dialysis was done in the dark and repeated three times yielding a total dilution of the dialysate by a factor of 10^6^. The evolution of the dialysis was followed by measuring the absorption spectrum of the dialysate. The same procedure was used to prepare RhBITC-BSA/PDA NPs, except that FITC was replaced by RhBITC. The concentrated solution of RhBITC (8.25 mM) was prepared by dissolving 4.4 mg in 1 mL of DMSO.

### Characterization of the BSA/polydopamine nanoparticles

#### Size measurement

The diameter of the NPs was measured by dynamic light scattering (DLS) using a Zetasizer Nano ZS from Malvern Panalytical (Malvern, UK). Measurements were performed while taking into account a refractive index of 1.73 − 0.02i for PDA (the imaginary part corresponds to the absorption coefficient) at a wavelength of 589 nm, that is, close to the wavelength of the laser used in the device (633 nm). Samples were diluted to get an absorption below 0.1. Size distribution results are given in intensity and can be expressed also in volume or number. Standard deviations of the sample’s mean hydrodynamic diameters as well as the polydispersity index are provided in Supporting Information File 1, Table S1. In this study, results given in number will be used.

#### Characterization of the fluorescence properties

Fluorescence spectra were recorded with a spectrophotometer-fluorimeter SAFAS Xenius XM 529 (SAFAS Monaco, Monaco, Monaco). Light absorption of BSA/PDA NPs, Ox-BSA/PDA NPs, FITC-BSA/PDA NPs, and RhBITC-BSA/PDA NPs was measured between 200 and 800 nm against Tris buffer. The fluorescence of Ox-BSA/PDA NPs, FITC-BSA/PDA NPs, and RhBITC-BSA/PDA NPs was characterized by emission spectra measurements at different excitation wavelengths in the range of 350–650 nm (photomultiplier voltage from 600 to 1000 V specified in the figure captions if needed; step of 1 or 5 nm). The emission intensity was compared with that of the fluorescent dyes, that is, FITC for FITC-BSA/PDA NPs and RhBITC for Ox-BSA/PDA NPs and RhBITC-BSA/PDA NPs. Solutions of free FITC and RhBITC were prepared in water from the FITC (3.2 mg/mL) and RhBITC (4.4 mg/mL) solutions in DMSO to provide concentrations of 164 and 373 µM, respectively. For the characterization of Ox-PDA/BSA-NPs, 5 µL of 120 µM RhBITC solution was added to 95 µL of 3 mg/mL Ox-PDA/BSA-NPs solution in Tris according to Ma and co-workers [21]. The curves were smoothened by the centered moving average method on three points. Finally, the fluorescence quantum yield of Ox-BSA/PDA NPs was determined by comparison with a DAPI reference (4 µM) according to the relative method [33]. For the measurements, Ox-BSA/PDA NPs and DAPI solutions were both made using Milli-Q^®^ water.

### Bacteriology

#### Bacterial species, strains, media, and culture conditions

Gram-negative *Escherichia coli* (*E. coli*) and Gram-positive *Staphylococcus aureus* (*S. aureus*) species were used for antibacterial testing: *E. coli* K-12 PHL628 (purchased from Prof. Philippe Lejeune, INSA-Lyon, France) [43] and *S. aureus* ATCC 25923 (purchased from Pasteur Institute, France). Lysogeny broth (LB) (Merck, Darmstadt, Germany) (pH 6.8) and Mueller–Hinton broth (MH) (Merck, Darmstadt, Germany) (pH 7.4) were prepared in distilled water and were sterilized by autoclaving at 121 °C for 30 min before use. Bacteria were thawed, diluted, plated on agar plates (LB for *E. coli* and MH for *S. aureus*), and incubated under aerobic conditions for 24 h at 37 °C.

#### Evaluation of the antibacterial activity

The antibacterial activity of pristine NPs, Ox-, and RhBITC-BSA/PDA NPs with a BSA/DA ratio of 10 was determined by calculating the bacterial growth inhibition from absorption measurements at 620 nm. Measurements were acquired with a Multiskan spectrophotometer (Fisher Scientific, Illkich, France). One colony of *E. coli* or *S. aureus* was transferred from the agar plate into fresh liquid LB (for *E. coli*) or MH (for *S. aureus*) and incubated at 37 °C overnight. The bacterial suspension was then diluted with fresh LB (for *E. coli*) or MH (for *S. aureus*) to 6 × 10^6^ CFU/mL. Lyophilized foam of BSA/PDA NPs with a BSA/DA ratio of 10 was diluted with 9 mg/mL NaCl solution to obtain a 20 mg/mL suspension of BSA/PDA NPs. This suspension was filtered in a microbiological safety cabinet with a 0.20 µm filter to eliminate possible contaminations and diluted in 9 mg/mL NaCl solution to prepare suspensions with NP concentrations of 0.5, 1, 2, 5, and 10 mg/mL. Finally, 10 µL of BSA/PDA NP suspension was added to 90 µL of bacterial solution (either *E. coli* or *S. aureus*) placed in 96 well plates to prepare bacterial suspensions with concentrations of BSA/PDA NPs from 0 to 2000 µg/mL (0 µg/mL (negative control C−), 50 µg/mL, 100 µg/mL, 200 µg/mL, 500 µg/mL, 1000 µg/mL, and 2000 µg/mL). A 10 µL mixture of tetracycline (10 µg/mL) and cefotaxime (0.1 µg/mL) was used as a positive control (C+) of bacterial growth inhibition. Bacterial growth was assessed from the absorption at 620 nm after 24 h of incubation at 37 °C. Each assay was performed three times. The statistical significance of two-by-two differences between the mean absorption at 620 nm was determined by unilateral Student’s tests. Bacterial growth inhibition was calculated for each concentration of NPs via Equation 4.


[4] $$\text{Inhibition }\left(\%\right)=\left(1-\frac{A_{\text{bact+NPs}}-A_{\text{NPs}}}{A_{\text{bact}}-A_{\text{LB}}}\right)\times 100,$$

where *A*_bact+NPs_, *A*_NPs_, *A*_bact_, and *A*_LB_ are the absorption values at 620 nm of the bacterial suspension containing NPs at a given concentration (*A*_bact+NPs_), the bacteria-free suspension containing NPs at a given concentration (*A*_NPs_), the bacterial suspension without NPs (*A*_bact_), and the suspension with neither bacteria nor NPs (*A*_LB_), respectively.

The minimal inhibitory concentration (MIC) corresponds to the lowest concentration of NPs that significantly inhibits growth of bacteria. If no inhibition effect is observed in the range of concentration tested, then the MIC will be indicated as higher than the maximum concentration tested.

#### Determination of the localization of NPs in bacterial cells

Standard and high-resolution fluorescence confocal laser scanning microscopy (CLSM) were used to determine the localization of the fluorescent BSA/PDA NPs with a BSA/DA ratio of 10 in bacterial cells. One colony of *E. coli* was transferred from the agar plate into fresh liquid LB and incubated at 37 °C overnight. The bacterial suspension was then diluted with LB to obtain a transmittance at 600 nm of 0.001 (6 × 10^6^ CFU/mL). 200 µL of fluorescent Ox-, FITC-, or RhBITC-BSA/PDA NPs suspension was added to 800 µL of the bacterial suspension, corresponding to the highest concentration of NPs tested in the previous assay, that is, 2 mg/mL of NPs. Three controls were also prepared: one with bacteria alone, one with NPs alone, and one with bacteria, without NPs but with fluorescent dye, either FITC or RhBITC, in a concentration equivalent to the quantity used to label the NPs. Because PDA absorbed a significant part of the light emitted by the fluorophores, the emission could only be roughly estimated by the difference between the intensity of the dye solution added to label BSA/PDA NPs and the intensity of the dialysis water. The equivalent dye concentrations (corresponding to 2 mg/mL of fluorescent NPs) were thus estimated to be 164 and 373 µM for FITC and RhBITC, respectively. The suspensions were all incubated at 37 °C for 24 h. Then, they were diluted in fresh liquid LB to reach a transmittance of 0.5 at 600 nm for suspensions without NPs, or a transmittance of 0.5 increased by the transmittance of the control with NPs alone, for samples containing NPs (to take into account the transmittance due to NPs). Afterward, samples were centrifuged at 3000 rpm for 10 min and re-suspended in 1 mL of PBS. This procedure was repeated two times. To allow for the storage of the samples over days, a fixation step was added to the preparation procedure. However, it is important to specify that this step is not required and that bacteria stained by the RhBITC- or FITC-BSA/PDA NPs can be observed alive directly after staining. The samples were fixed with paraformaldehyde (PFA): Samples were centrifuged at 3000 rpm for 10 min, re-suspended in 500 µL PBS 2× and 500 µL of PFA 8%, then centrifuged again at 3000 rpm for 10 min and re-suspended and incubated for 1 h in PFA 4% in PBS 1×. Samples were washed by one last cycle of centrifugation (3000 rpm, 10 min) and re-suspension in PBS (1 mL). 8 µL of the suspension was finally deposited on a glass slide and covered by a glass coverslip, both previously washed using 0.2% Hellmanex^®^ cleaning solution and ethanol in an ultrasonic bath. After complete drying (15 min), 8 µL of mounting medium (ProLong™ Diamond, Molecular Probes™) was deposited on the dry drop of sample, before adding a cover glass and sealing it with nail polish. The samples were kept at 4 °C in the darkness.

Samples were observed with a standard CLSM Zeiss LSM710 (Zeiss, Oberkochen, Germany) equipped with a 63× objective lens (X63 PL APO oil/1.4) and a high-resolution confocal microscope Stellaris 5 (Leica Biosystems, Wetzlar, Germany) equipped with a 100× objective lens (X100 HCX PL APO oil/1.4). Using the LSM710 microscope, excitation and emission wavelengths were 405, 488, and 561 nm, and in the ranges of 460–541, 495–634, and 567–703 nm, respectively. Using the Stellaris microscope, excitation and emission wavelengths were 405, 488, and 543 nm, and in the ranges of 415–482, 496–565, and 553–628 nm, respectively. With the Stellaris microscope, 3D-stack images with thicknesses from 1.5 to 5.0 µm with steps from 125 to 145 nm were recorded to allow for the localization of fluorescence emission related to the bacterial cells. Eight different types of samples were prepared, which are described in Table 2.

**Table 2:** Samples prepared for fluorescence confocal microscopy.

Sample composition	*E. coli* concentration (CFU/mL)	NP concentration (mg/mL)	Fluorescent dye concentration (in NPs or free) (µM)

BSA/PDA NPs + *E. coli*	6 × 10^6^	2	0
Ox- BSA/PDA NPs + *E. coli*	6 × 10^6^	2	0
FITC-BSA/PDA NPs + *E. coli*	6 × 10^6^	2	164^a^
RhBITC-BSA/PDA NPs + *E. coli*	6 × 10^6^	2	373^a^
BSA/PDA NPs	0	2	0
Ox-BSA/PDA NPs	0	2	0
FITC- BSA/PDA NPs	0	2	164^a^
RhBITC-BSA/PDA NPs	0	2	373^a^
FITC + *E. coli*	6 × 10^6^	0	164
RhBITC + *E. coli*	6 × 10^6^	0	373
*E. coli*	6 × 10^6^	0	0

^a^Estimation based on the difference in absorption intensity of the fluorescent dye solution used to label BSA/PDA NPs and the absorption intensity of the dialysis water.

## Supporting Information

Supporting Information File 1 provides additional tables and figures regarding synthesis, characteristics, and properties (including accumulation in bacterial cells) of pristine and fluorescent BSA/PDA NPs.

File 1 Additional tables and figures.
