# Supplementary material for: Fluorescent bioinspired albumin/polydopamine nanoparticles and their interactions with Escherichia coli cells
Source: Beilstein J Nanotechnol. 2023 Dec 22;14:1208–24. doi: 10.3762/bjnano.14.100 (PMC10760463; doi:10.3762/bjnano.14.100)
Supplement: File 1 — Additional tables and figures. [file Beilstein_J_Nanotechnol-14-1208-s001.pdf]

BEILSTEIN JOURNAL OF NANOTECHNOLOGY

Supporting Information

for

Fluorescent bioinspired albumin/polydopamine nanoparticles and their interactions with *Escherichia coli* cells

Eloïse Equy, Jordana Hirtzel, Sophie Hellé, Béatrice Heurtault, Eric Mathieu, Morgane Rabineau, Vincent Ball and Lydie Ploux

Beilstein J. Nanotechnol. **2023,** *14,* 1208–1224. doi:10.3762/bjnano.14.100

Additional tables and figures

License and Terms: This is a supporting information file under the terms of the Creative Commons Attribution License (https://creativecommons.org/licenses/by/4.0). Please note that the reuse, redistribution and reproduction in particular requires that the author(s) and source are credited and that individual graphics may be subject to special legal provisions.
The license is subject to the *Beilstein Journal of Nanotechnology* terms and conditions: (https://www.beilstein-journals.org/bjnano/terms)

Table S1: Mean hydrodynamic diameter and polydispersity index (PDI) of BSA/PDA NPs in Tris buffer 50 mM (without dialysis and freeze-drying).

	Ratio BSA/DA	Mean diameter in intensity (nm)	Mean diameter in number (nm)	PDI
BSA/PDA-NPs	0.25	83 ± 22	66 ± 16	0.21 ± 0.01
	1	32 ± 7	26 ± 6	0.32 ± 0.06
	3	18 ± 5	14 ± 3	0.27 ± 0.04
	6	15 ± 4	11 ± 3	0.27 ± 0.04
	10	15 ± 7	9 ± 3	0.27 ± 0.04
Ox-BSA/PDA-NPs	10	15 ± 4	11 ± 3	ND
RhBITC-BSA/PDA-NPs	10	28 ± 6	24 ± 3	0.57 ± 0.06
FITC BSA/PDA-NPs	10	17 ± 5	12 ± 3	0.19 ± 0.01

Table S2: Concentration, number of NPs per mL, and reaction yield of the as-prepared pristine BSA/PDA NPs solutions.

Ratio BSA/DA	Concentration (mg/mL)	Number of NPs /mL	Reaction Yield (%)
0.25	2	5×10^{15}	85
1	2	8×10^{16}	50
3	3	2×10^{14}	85
6	9	3×10^{18}	63
10	19	4×10^{15}	85

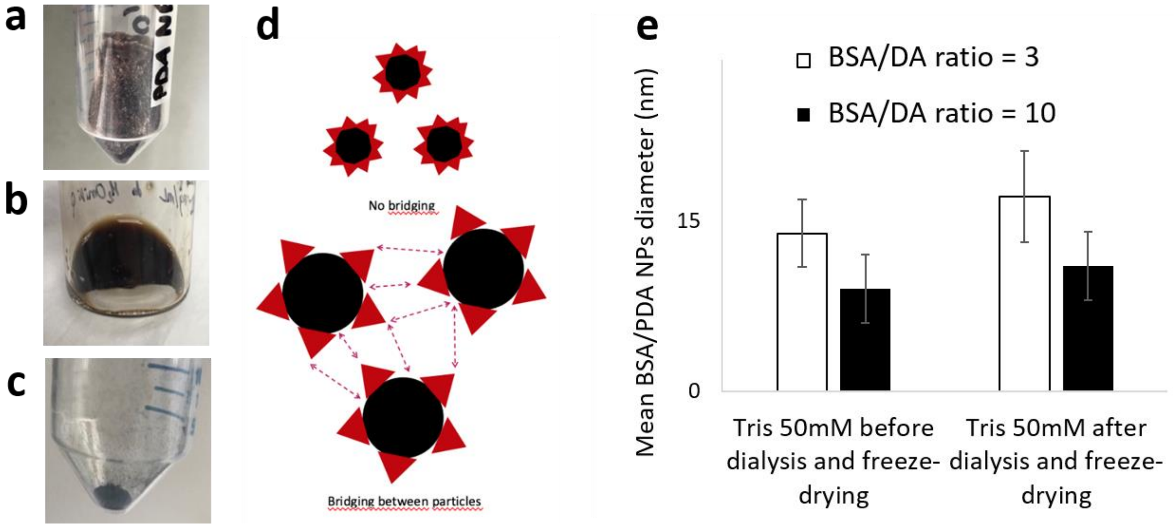

Figure S1: BSA/PDA NPs after freeze-drying. Pictures of (a) a foam obtained after freeze-drying of a BSA/PDA NPs suspension synthesized with a BSA/DA ratio of 3. (b) PDA/BSA NPs re-dissolved foam into Milli-Q® water (10mg/mL). (c) PDA/BSA-NPs ratio of 0.25 dehydrated powder. (d) Schematic of bridging between PDA/BSA-NPs. (e) PDA/BSA-NPs size variation due to dialysis and freeze-drying.

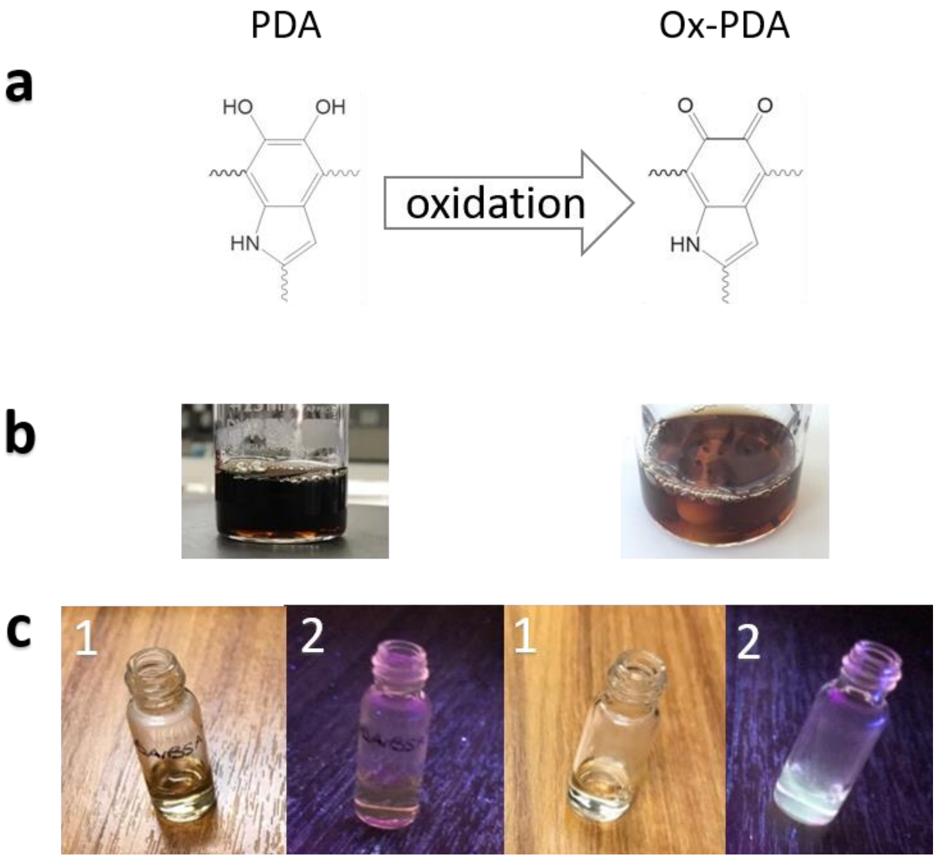

Figure S2: Oxidation of pristine BSA/PDA NPs providing fluorescent Ox-BSA/PDA NPs. (a) Reaction scheme of PDA (inspired from Ma et al. [1]). (b) Pictures of PDA/BSA-NPs suspensions after 1 h (left) or 24 h (right) of reaction with H_2O. (c) Pictures of PDA/BSA-NPs suspensions before (left) and after (right) oxidation (1) under natural light and (2) under UV light (365 nm).

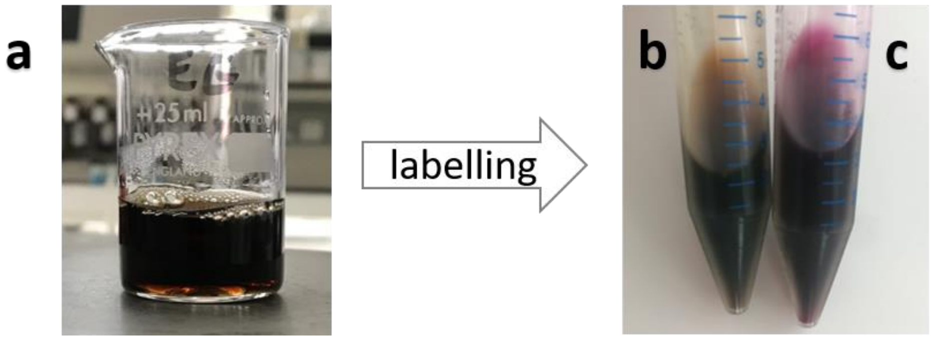

Figure S3: Pictures of FITC- and RhBITC-BSA/PDA NPs suspensions after dialysis with a 8–10 kDa cut-off membrane. (a) FITC-BSA/PDA NPs. (b) RhBITC-BSA/PDA NPs (c).

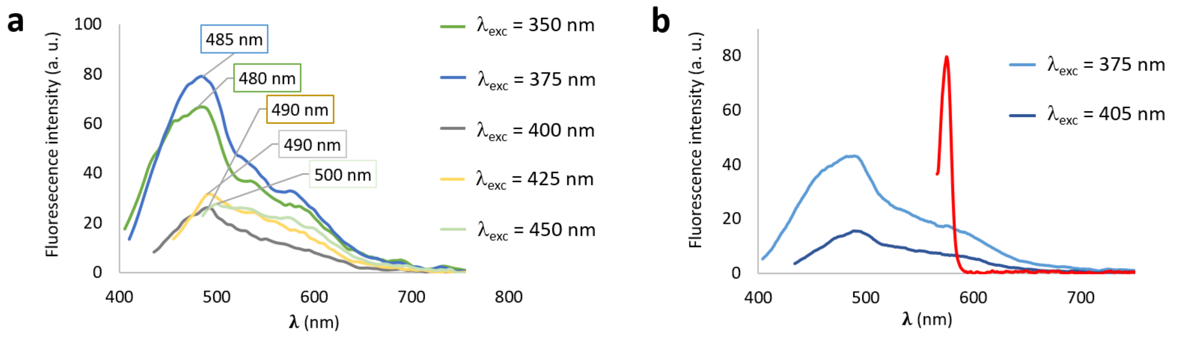

Figure S4: Absorption and emission spectra of Ox-PDA/BSA-NPs. (a) Emission spectra according to various excitation wavelengths (λ_{exc}) (PMT voltage at 1000 V); wavelengths corresponding to fluorescence intensity maxima are indicated in labels; spectra were smoothed by moving average method on 3 points. (b) Emission spectra of a mixture of Ox-PDA/BSA-NPs (3 mg/mL) and RhBITC (0.06 mg/mL) (95/5) under different excitation wavelengths (λ_{exc}) (PMT voltage at 900 V); spectra were smoothed by centered moving average method on 9, 9 and 3 points, respectively.

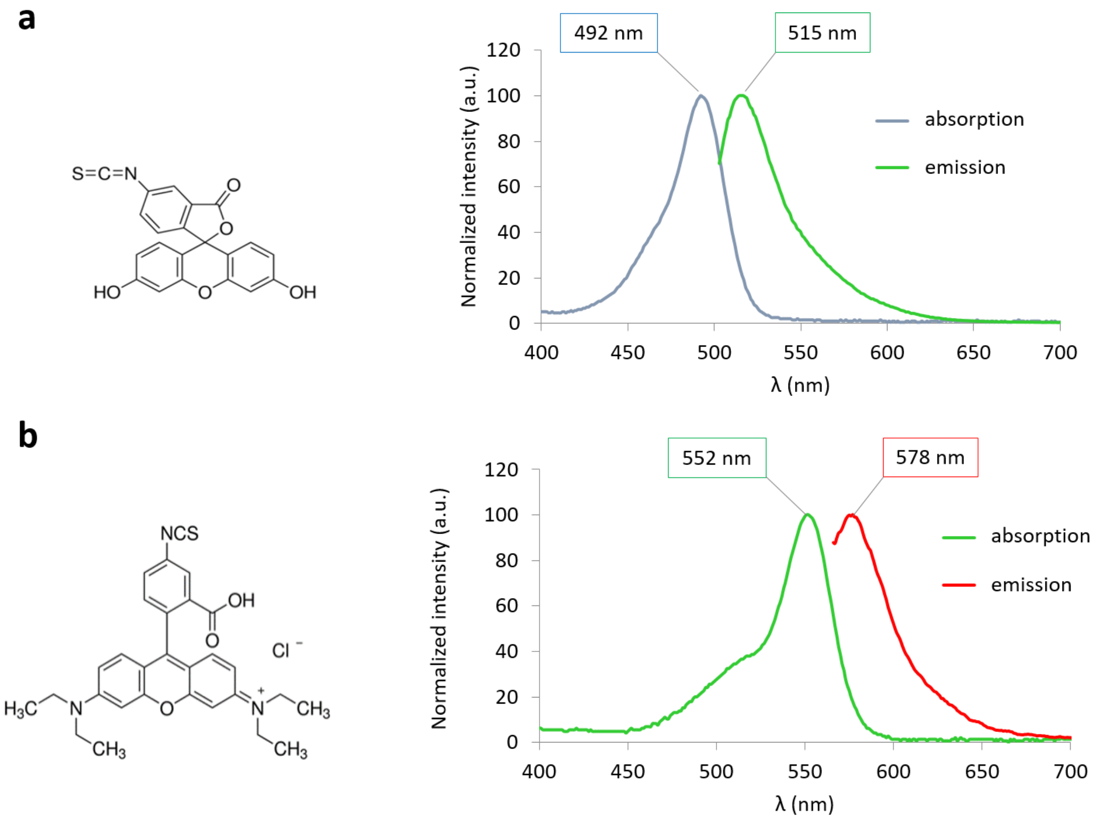

Figure S5: Absorption and emission spectra of free FITC and RhBITC. (a) Structure and normalized absorption and emission spectra of free FITC (164 µM) under excitation wavelength λ_{exc} of 488 nm. (b) Structure and normalized absorption and emission spectra of free RhBITC (373 µM) under excitation wavelength λ_{exc} of 550 nm. Spectra were smoothed by centered moving average method on 3 points.

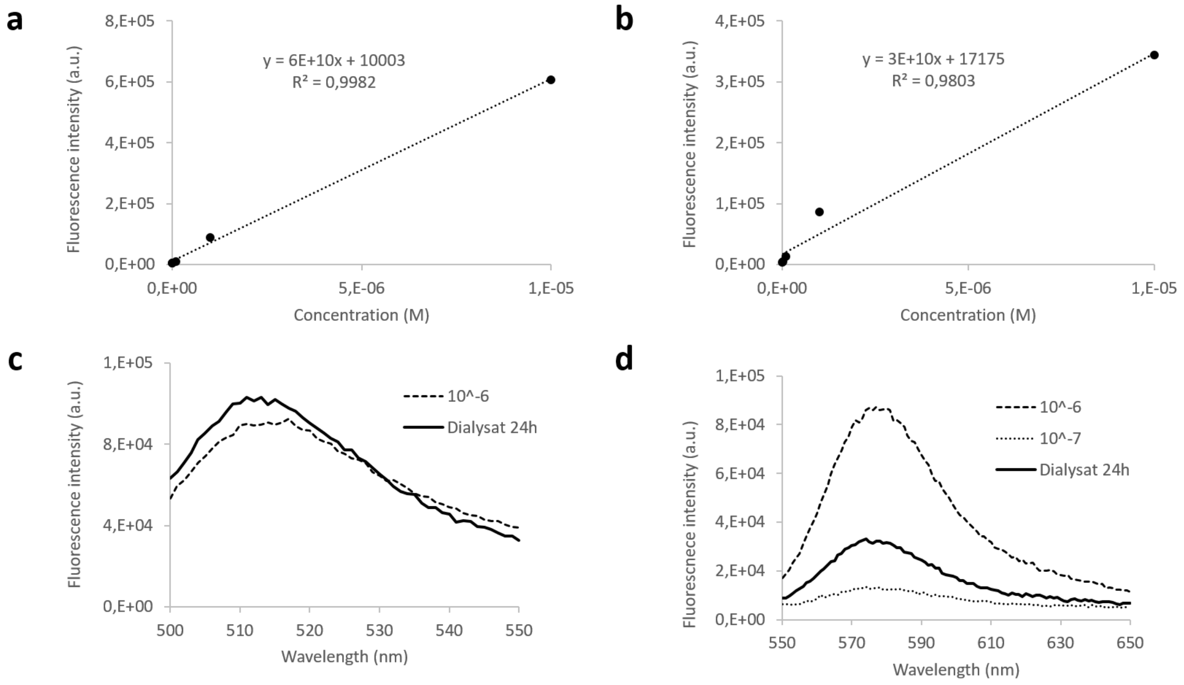

Figure S6: Estimation of the fraction of bound dye released from FITC-BSA/PDA NPs and RhBITC-BSA/PDA NPs after 4 months. (a), (b) The relationships between the fluorescence intensity and dye concentration were established for both FITC (a) and RhBITC (b) dyes. (c), (d) Dialysis was conducted with labelled NPs against 2 L of Milli-Q® water using a Spectra/Por® dialysis membrane with a molecular weight cut-off of 12–14 kDa. Based on the dye concentrations estimated to initially bind to NPs (164 µM of FITC and 373 µM of RhBITC), less than 1 % of dye (1.5 µM -0.9%- for FITC and 0.5 µM -0.1%- of RhBITC) was estimated to release from 4-months old labelled NPs.

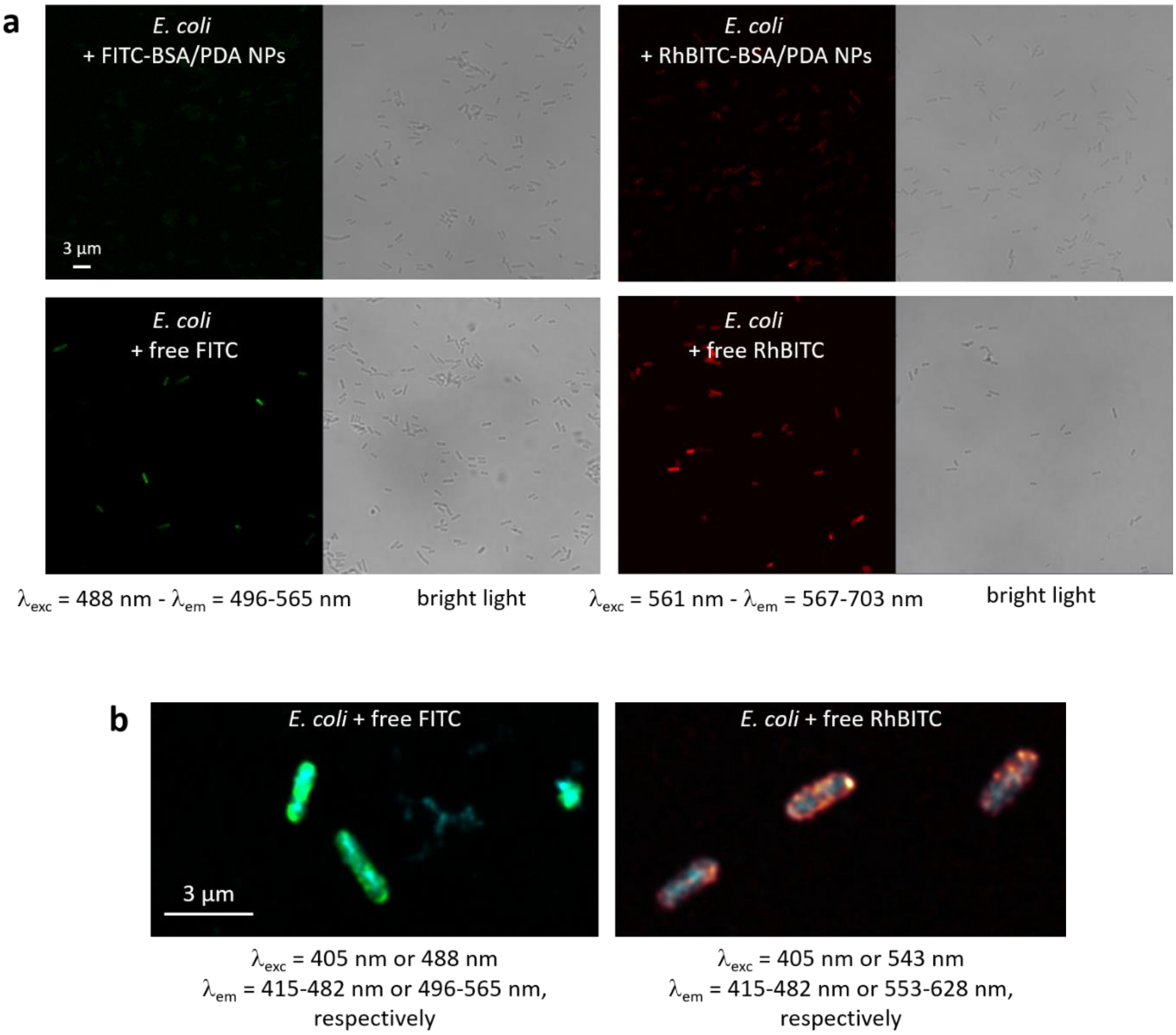

Figure S7: Micrographs of *E. coli* cells cultivated (a) with FITC-BSA/PDA NPs, RhBITC-BSA/PDA NPs, free FITC or free RhBITC and observed with standard CLSM (LSM710, Zeiss, Oberkochen, Germany), or (b) with free FITC or free RhBITC and observed with high-resolution CLSM (Stellaris 5, Leica Biosystems, Wetzlar, Germany).

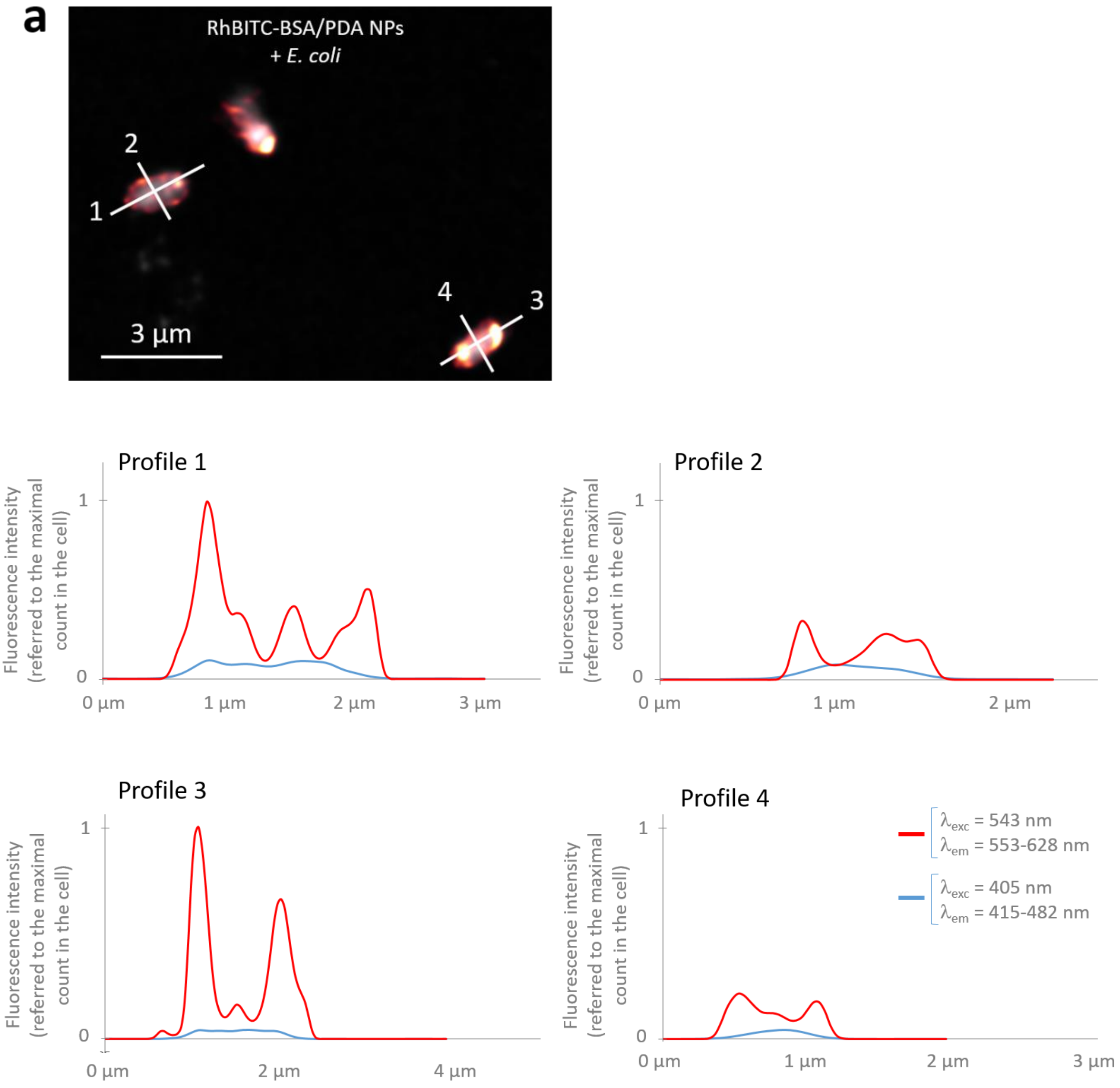
a
RhBITC-BSA/PDA NPs
+ E. coli
1
2
3
4
3 µm
Profile 1
Profile 2
Profile 3
Profile 4
Fluorescence intensity
(referred to the maximal
count in the cell)
0
1
0 µm
1 µm
2 µm
3 µm
4 µm
λexc = 543 nm
λem = 553-628 nm
λexc = 405 nm
λem = 415-482 nm

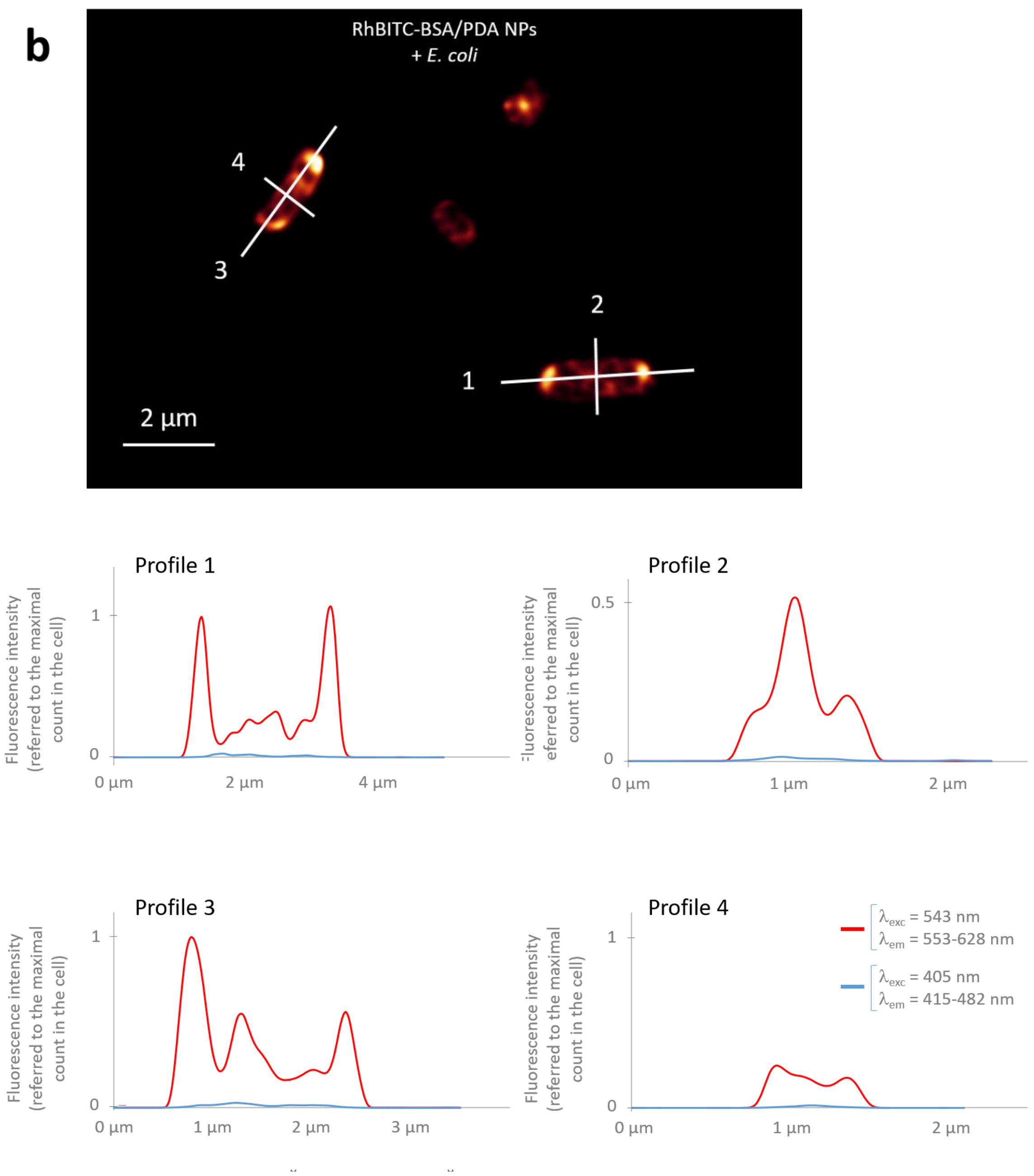
b
RhBITC-BSA/PDA NPs
+ E. coli
1
2
3
4
2 µm
Profile 1
Profile 2
Profile 3
Profile 4
Fluorescence intensity (referred to the maximal count in the cell)
0 µm
1 µm
2 µm
3 µm
4 µm
0
0.5
1
λexc = 543 nm
λem = 553-628 nm
λexc = 405 nm
λem = 415-482 nm

c

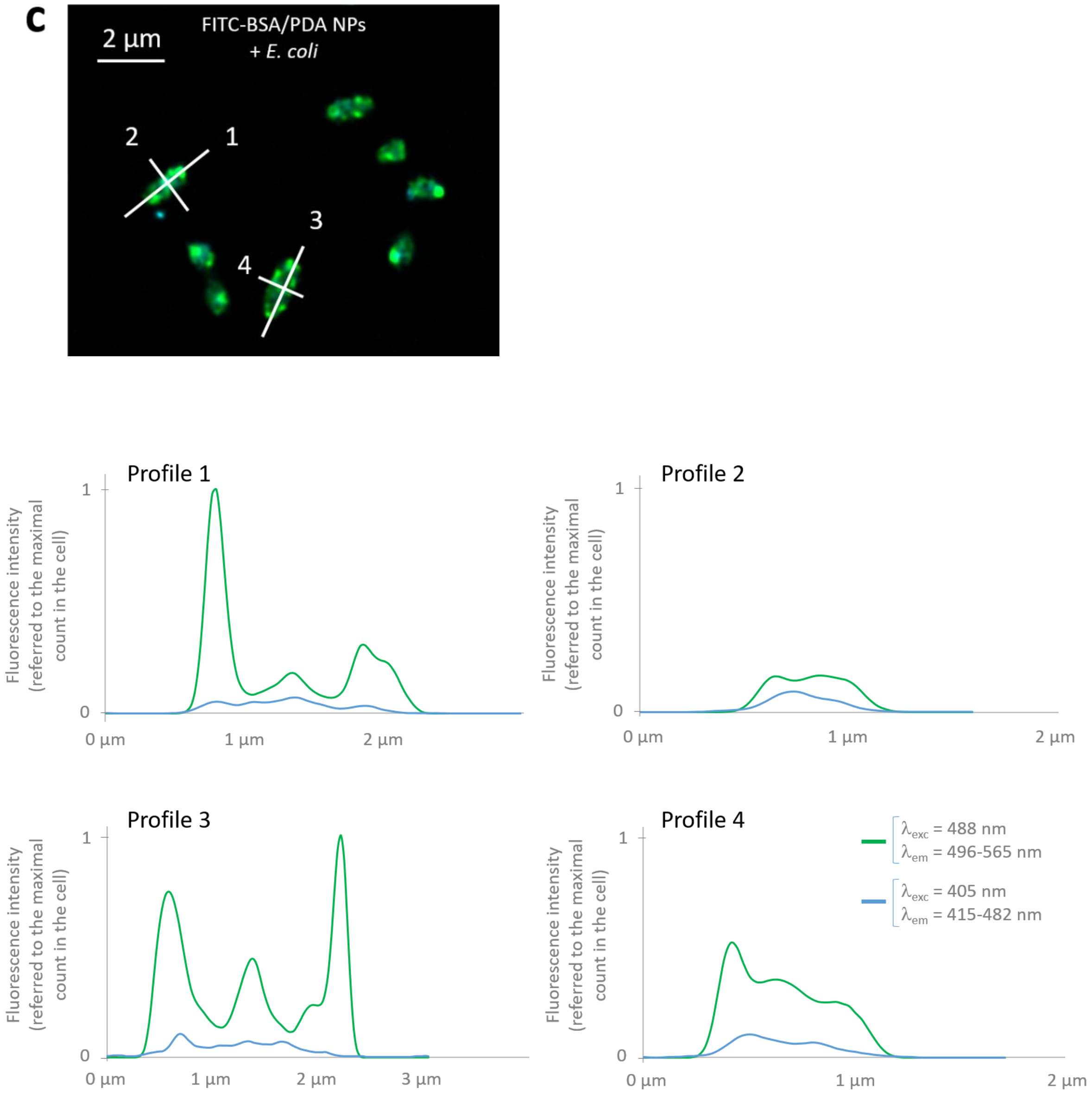

2 µm
FITC-BSA/PDA NPs
+ *E. coli*
1
2
3
4
Profile 1
Profile 2
Profile 3
Profile 4
Fluorescence intensity
(referred to the maximal
count in the cell)
0
1
0 µm
1 µm
2 µm
3 µm
λexc = 488 nm
λem = 496-565 nm
λexc = 405 nm
λem = 415-482 nm

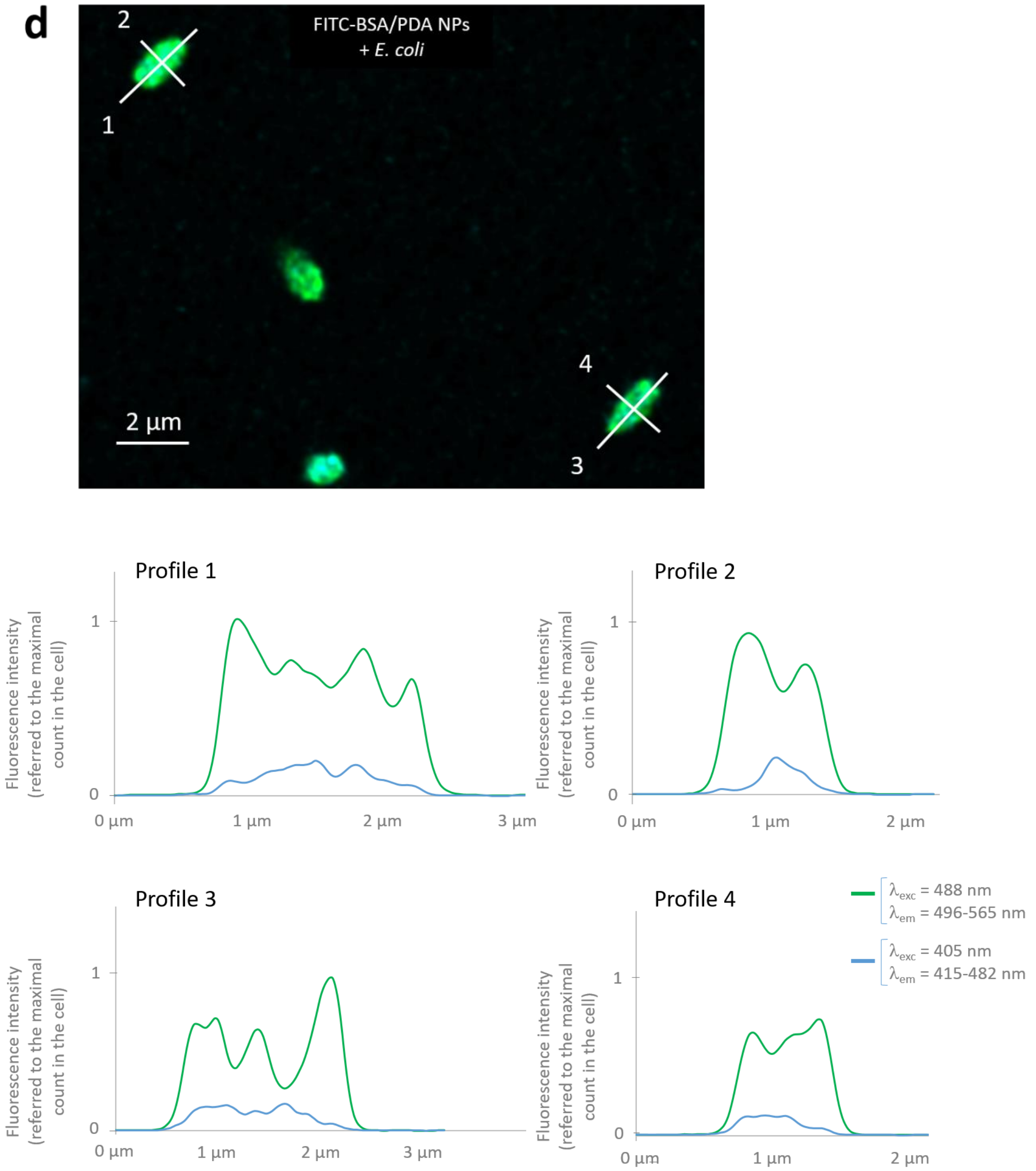

Figure S8: Typical profiles and patterns observed in micrographs of *E. coli* cells cultivated with RhBITC- or FITC-BSA/PDA NPs observed by high-resolution Stellaris 5 CLSM (Leica Biosystems, Wetzlar, Germany): (a) and (b) Two supplementary, typical examples of bacterials cells cultivated with RhBITC-BSA/PDA NPs (λ_{exc} = 405 nm and 543 nm; λ_{em} = 415–482 nm and 553–628 nm, respectively); (c) and (d) Two typical examples of bacterial cells cultivated with FITC-BSA/PDA NPs (λ_{exc} = 405 nm and 488 nm; λ_{em} = 415–482 nm and 496–565 nm, respectively).

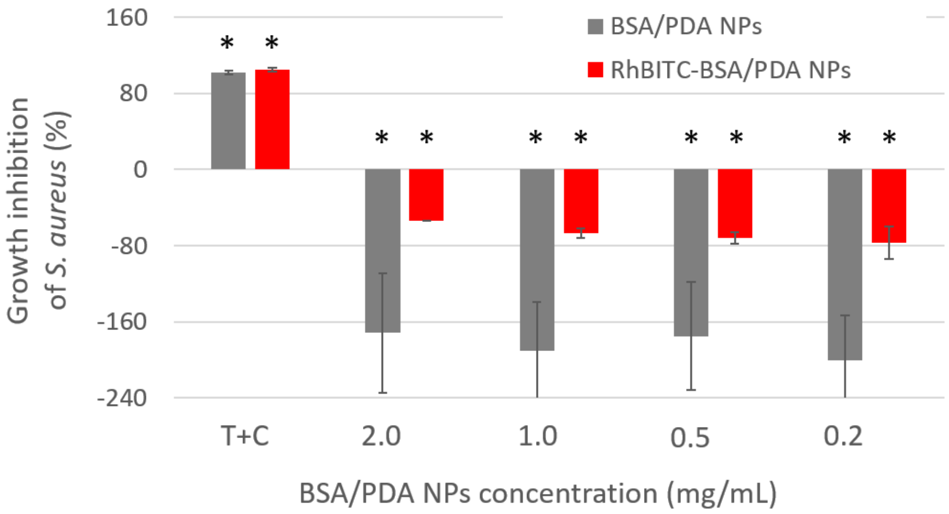

Figure S9: Growth inhibition of *S. aureus* populations with pristine BSA/PDA NPs and RhBITC-BSA/PDA NPs from 0.2 to 2 mg/mL concentration or with antibiotics (solution of 10 µg/mL tetracycline and 0.1 µg/mL cefotaxime) ("T+C"; positive control) compared to *S. aureus* culture without NPs. **: significant differences to *S. aureus* culture without NPs (*p*-value < 0.001).

References

1. Ma, B.; Liu, F.; Zhang, S.; Duan, J.; Kong, Y.; Li, Z.; Tang, D.; Wang, W.; Ge, S.; Tang, W.; Liu, H. *Journal of Materials Chemistry B* **2018**, *6* (40), 6459-6467. doi:10.1039/C8TB01930D
